# Multiplexed drug-based selection and counterselection genetic manipulations in *Drosophila*

**DOI:** 10.1016/j.celrep.2021.109700

**Published:** 2021-09-14

**Authors:** Nick Matinyan, Mansi S. Karkhanis, Yezabel Gonzalez, Antrix Jain, Alexander Saltzman, Anna Malovannaya, Alejandro Sarrion-Perdigones, Herman A. Dierick, Koen J.T. Venken

**Affiliations:** 1 Verna and Marrs McLean Department of Biochemistry and Molecular Biology, Baylor College of Medicine, Houston, TX 77030, USA; 2 Integrative Molecular Biomedical Sciences Graduate Program, Baylor College of Medicine, Houston, TX 77030, USA; 3 Department of Molecular and Human Genetics, Baylor College of Medicine, Houston, TX 77030, USA; 4 Advanced Technology Cores, Mass Spectrometry Proteomics, Baylor College of Medicine, Houston, TX 77030, USA; 5 Dan L. Duncan Comprehensive Cancer Center, Baylor College of Medicine, Houston, TX 77030, USA; 6 Department of Neuroscience, Baylor College of Medicine, Houston, TX 77030, USA; 7 Department of Pharmacology and Chemical Biology, Baylor College of Medicine, Houston, TX 77030, USA; 8 McNair Medical Institute at The Robert and Janice McNair Foundation, Baylor College of Medicine, Houston, TX 77030, USA; 9 Present address: Solomon H. Snyder Department of Neuroscience, Johns Hopkins University School of Medicine, Baltimore, MD 21205, USA; 10 These authors contributed equally; 11 Twitter: @Bio_TECH_niC; 12 Lead contact

## Abstract

The power of *Drosophila melanogaster* as a model system relies on tractable germline genetic manipulations. Despite *Drosophila*’s expansive genetics toolbox, such manipulations are still accomplished one change at a time and depend predominantly on phenotypic screening. We describe a drug-based genetic platform consisting of four selection and two counterselection markers, eliminating the need to screen for modified progeny. These markers work reliably individually or in combination to produce specific genetic outcomes. We demonstrate three example applications of multiplexed drug-based genetics by generating (1) transgenic animals, expressing both components of binary overexpression systems in a single transgenesis step; (2) dual selectable and counterselectable balancer chromosomes; and (3) selectable, fluorescently tagged *P[acman]* bacterial artificial chromosome (BAC) strains. We perform immunoprecipitation followed by proteomic analysis on one tagged BAC line, demonstrating our platform’s applicability to biological discovery. Lastly, we provide a plasmid library resource to facilitate custom transgene design and technology transfer to other model systems.

## In brief

Matinyan et al. describe a set of four drug selection and two drug counterselection markers for use in *Drosophila* genetics. The orthogonal markers can be used individually or combined for complex, multiplex genetic manipulations. Use of drug selection and counterselection eliminates screening efforts for modified animals, saving time and cost.

## Graphical Abstract

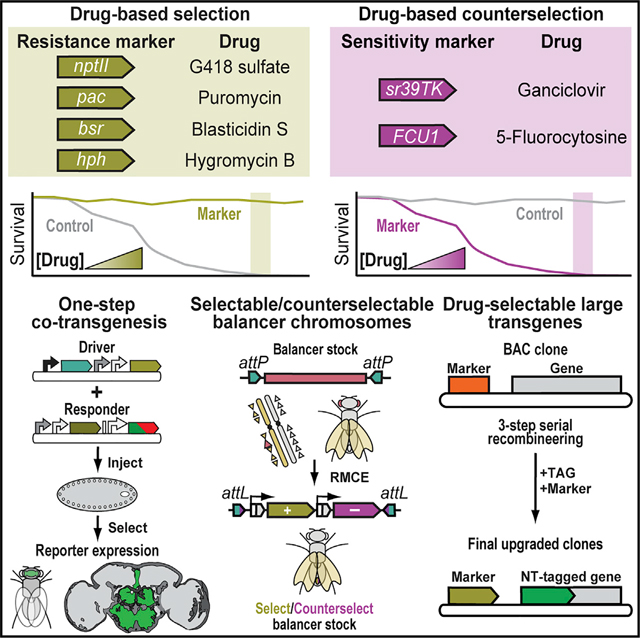

## INTRODUCTION

Genetic engineering technologies in *Drosophila melanogaster* have greatly advanced the study of basic biology and human disease (Bier, 2005; Venken and Bellen, 2007, 2014; Venken et al., 2016; Bellen et al., 2019; Link and Bellen, 2020). Historically, germline genetic manipulations in fruit flies, e.g., insertional mutagenesis and transgenesis, almost exclusively relied on P element transposons (Ryder and Russell, 2003). They were either remobilized from one location in the genome to another (Cooley et al., 1988) or jumped into the genome from a microinjected plasmid (Rubin and Spradling, 1982). Transposon-based mutagenesis and transgenesis have largely been replaced by CRISPR-Cas9-mediated genome editing (Gratz et al., 2014; Bier et al., 2018) and ΦC31-mediated, site-specific integration (Groth et al., 2004; Bischof et al., 2007), respectively.

Introducing tractable genetic modifications into flies starts with microinjecting a plasmid (Venken and Bellen, 2007). This plasmid carrying a transgene coupled to a dominant physical marker is injected into early-stage, syncytial embryos targeting the future germline in a genetic background deficient for the marker (Venken and Bellen, 2007). Adult flies that survive the injection process may have transformed germ cells that upon crossing will produce offspring identifiable by marker expression (Figure S1A). The most commonly used dominant markers are the eye color markers, *white^+^* and *vermillion^+^*, and the body color marker, *yellow^+^* (Venken and Bellen, 2007). Screening genetically modified progeny can be time consuming and laborious (Venken et al., 2006, 2009, 2010; Venken and Bellen, 2007; Dahmann, 2008; Beumer and Carroll, 2014; Bier et al., 2018).

To eliminate the workload of screening, a drug-based selection approach can be used instead (Figure S1B; Steller and Pirrotta, 1985; Handler and O’Brochta, 1991; Semple et al., 2010; Giordano-Santini and Dupuy, 2011; Kandul et al., 2020). Selection markers confer resistance to universally toxic antibiotics, killing non-transgenic progeny but allowing transgenic, drug-resistant animals to survive (Steller and Pirrotta, 1985). As these markers are exogenous to the fly, drug-based selection can be used in any genetic background (Steller and Pirrotta, 1985), in contrast to many physical screening markers that require marker null allele genotypes (Venken and Bellen, 2007). Fluorescent markers are also exogenous and dominantly expressed, but they still require screening and can interfere with downstream analyses (e.g., fluorescence microscopy; Horn et al., 2002; Venken and Bellen, 2007). Selectable markers have been most successfully used in the nematode *Caenorhabditis elegans*, where several exogenous drug-resistance markers have been found effective (Giordano-Santini et al., 2010; Semple et al., 2010; Radman et al., 2013; Kim et al., 2014). In the fruit fly, drug-based selection was implemented shortly after the advent of P-element-based transgenesis (Rubin and Spradling, 1982; Steller and Pirrotta, 1985) but was largely abandoned due to variability of marker-conferred drug resistance, likely due to the inherent randomness of P element transposon integration (Steller and Pirrotta, 1985; Handler and O’Brochta, 1991). Selection strategies using orthogonal selectable marker genes would greatly expand the utility of this technology and provide opportunities for multiplexed selectable genetic engineering applications.

Similarly, counterscreening against an undesired outcome of a genetic manipulation depends on dominant physical or fluorescent markers. These are coupled to an unwanted genotype, and counterscreening against them can be equally laborious as screening (Figure S2A). Drug sensitivity markers eliminate counterscreening by counterselecting against those genotypes using the relevant counterselection agent instead (Figure S2B). Drug-sensitized animals will not survive, whereas non-sensitive progeny will be unaffected by drug exposure. *In vivo* animal counterselection, akin to counterselection in bacteria and cell culture, has been primarily used for efforts to control disease vector and agricultural pest insect populations by biasing genetic sexing (i.e., generation of purely male populations; Thomas et al., 2000; Flores and O’Neill, 2018; Kandul et al., 2020). Of the three most notable examples, two of the approaches use a two-component tetracycline-repressible expression system to drive female-specific expression of the proapoptotic gene *head involution defective* (*hid*) (Heinrich and Scott, 2000; Thomas et al., 2000). A third approach uses female-specific expression of the bacterial suicide gene *cytosine deaminase* (*codA*), sensitizing females to the nucleoside analog 5-fluorocytosine (Markaki et al., 2004). However, *in vivo* counterselection is not yet part of the basic genetic toolbox in *Drosophila*. Previous efforts have shown the efficacy of counterselectable chromosomes by coupling the cell death gene *reaper* (*rpr*) to a *UAS* element, which if crossed to a *GAL4* source will result in the death of the resulting progeny (McMahan et al., 2013). However, this system precludes the use of the *GAL4* binary expression system for other applications. Counterselection strategies using orthogonal counterselectable marker genes would further expand the utility of this application and make multiplex counterselectable genetic manipulations possible.

We describe a drug-based selection and counterselection platform for multiplexed genetic manipulations in *Drosophila melanogaster* that is readily transferrable to other model systems. We designed a compact expression cassette to test five drug-resistance and two drug-sensitivity markers. We successfully select drug-resistant transgenic animals with four of the five resistance markers using the drugs G418 sulfate, puromycin, blasticidin S, and hygromycin B. We also demonstrate effective counterselection against drug-sensitized animals using either ganciclovir or 5-fluorocytosine. By showing that marker-conferred drug resistance or sensitivity is specific to the corresponding drug, we combine multiple drugs in single, multiplexed genetic manipulations to perform co-selection, combination selection and counterselection, and co-counterselection, generating distinct genotypes. We also apply this platform to make double-transgenic animals in a single step, generate selectable and counterselectable balancer chromosomes, and make fluorescently tagged selectable *P[acman]* transgenics. We use one of the *P[acman]* transgenics to establish an unbiased interactome, demonstrating applicability of our platform for biological discovery. Finally, we provide a vector library resource to allow easy adoption of our selection and counterselection genetic platform, facilitating designs for custom applications as well as for adaptation to other model and non-model species.

## RESULTS

### Determining effective drug concentrations and specificity of selection and counterselection markers

**Figure 1.**
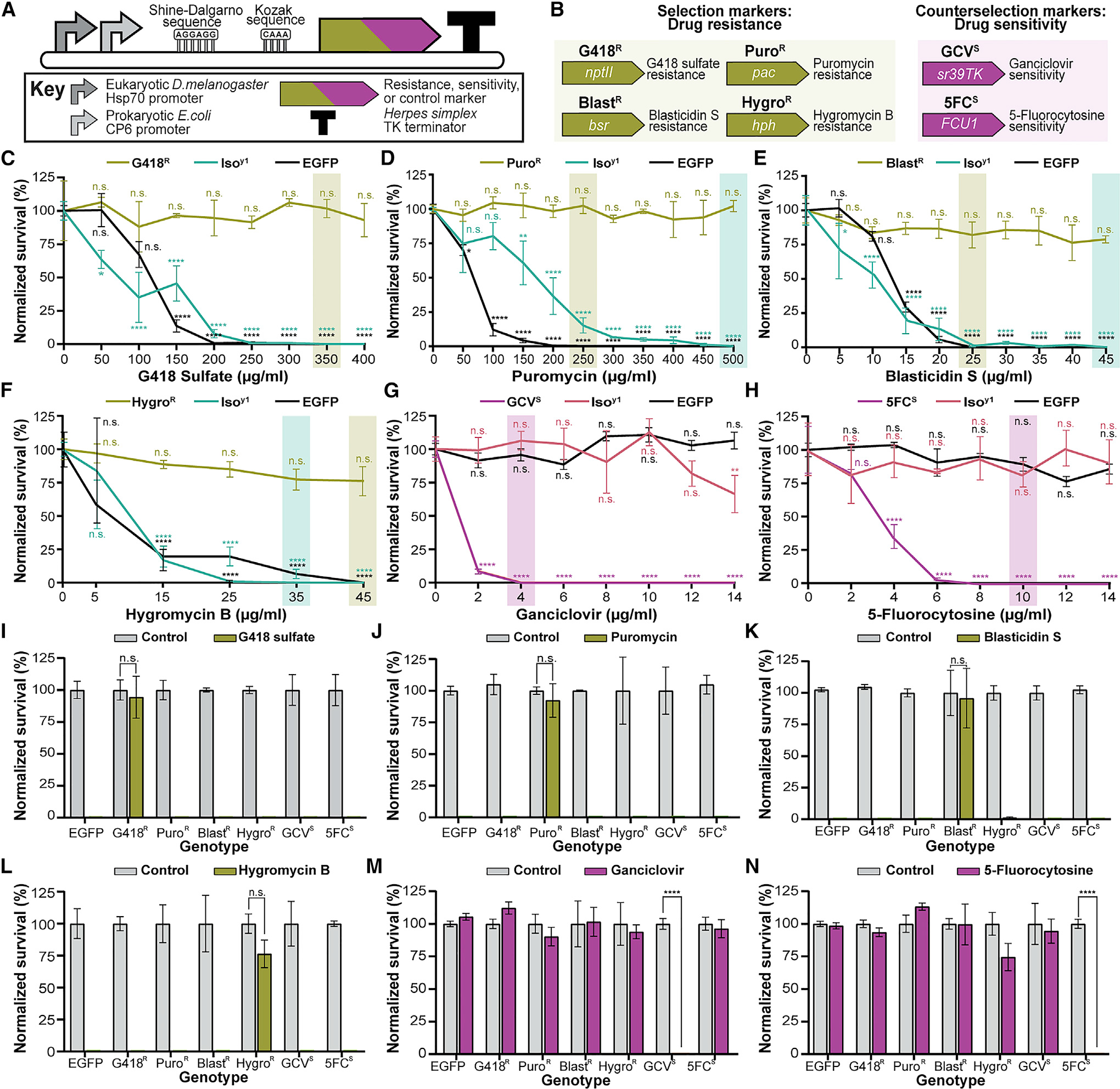
Determining effective drug concentrations and specificity of selection and counterselection markers (A) Schematic of the marker expression cassette. The fusion promoter works in bacteria and flies. Marker expression can be enhanced in flies via heat shock. Expression-enhancing elements are indicated: a bacterial Shine-Dalgarno; a consensus *Drosophila* Kozak/Cavener sequence; and a TK poly(A) terminator. (B) Schematic summary of markers (italics) and their corresponding drug resistance or sensitivity and strains (bold). (C–H) Determining effective selection and counterselection concentrations for drug-based selection and counterselection in two genetic backgrounds. (C–F) The effective selection concentration (ESC) is the concentration of drug at which the control, EGFP-expressing strain is eliminated while the drug-resistant strain is unaffected. ESC for G418 sulfate is 350 μg/mL (G418^R^) (C), 250 or 500 μg/mL for puromycin (Puro^R^) (D), 25 or 45 μg/mL for blasticidin S (Blast^R^) (E), and 65 or 45 μg/mL for hygromycin B (Hygro^R^) (F). (G and H) The effective counterselection concentration (ECC) is the drug concentration at which the sensitivity-marker-expressing strain is eliminated while the control EGFP-expressing fly strain survival is unaffected versus vehicle control. ECC for ganciclovir is 4 μg/mL (GCVS) (G) and 10 μg/mL for 5-fluorocytosine (5FCS) (H). (I–L) Only correspondingly resistant fly strains survive drug treatment at determined ESC (see C–F) for G418 sulfate (I), puromycin (J), blasticidin S (K), and hygromycin B (L). (M and N) Only the corresponding sensitive strain survival is affected by treatment with ganciclovir (M) or 5-fluorocytosine (N) at their respective ECC (Table 1). Statistical significance for the survival curves was determined via two-way ANOVA using Dunnett’s multiple comparisons test for each strain on each drug compared to vehicle survival. Statistical significance of marker orthogonality was determined via multiple t test between untreated and treated vials of the same strain for each drug using the Holm-Sidak method. For both methods, α = 0.05, *p < 0.05, **p < 0.01, ***p < 0.001, ****p < 0.0001, n.s. is non-significant, and data shown represent mean (Dunnett) or average (Holm-Sidak) and SEM for at least three biological replicates per condition. See also Figures S1–S3.

We developed a compact expression cassette using the same marker expression in bacteria for molecular cloning and in *Drosophila* for transgenesis (Figure 1A; see STAR Methods for details). We cloned five selection markers, two counterselection markers, and an EGFP control marker into our cassette (Table 1; Figure 1B). The five selection markers were neomycin phosphotransferase II (*nptII*), puromycin N-acetyltransferase (*pac*), blasticidin S-resistance (*bsr*), hygromycin B phosphotransferase (*hph*), and bleomycin resistance protein (*ble*). Their corresponding drugs are G418 sulfate, puromycin HCl, blasticidin S, hygromycin B, and zeocin or phleomycin. The two counterselection markers were thymidine kinase (*sr39TK*) and fusion protein of cytosine deaminase and uracilphosphoribosyltransferase (FCU1), used with ganciclovir and 5-fluorocytosine, respectively. We made transgenic fly strains for each marker, resulting in *G418^R^*, *Puro^R^*, *Blast^R^*, *Hygro^R^*, *Zeo^R^*, *GCV^S^*, *5FC^S^*, and *EGFP* flies, all inserted into the same genomic locus (Table 1; Figure 1B).

We next examined the survival of resistant, sensitive, and control marker strain flies on food treated with varying drug concentrations to determine their respective effective selection concentration (ESC) or effective counterselection concentration (ECC) (Figures 1C–1H). The ESC is the lowest drug concentration that eliminates all non-resistant flies without significantly affecting survival of the drug-resistant flies. Conversely, the ECC is the lowest drug concentration that eliminates all sensitized animals without significantly affecting control background fly survival. We carried out similar dose-response experiments using an isogenized fly strain (*Iso^Y1^*) to determine the ESC and ECC dependency on genetic background (Figures 1C–1H). Except for the *ble* marker (*Zeo^R^*; Figure S3), we successfully determined an ESC or ECC for all drugs in both genetic backgrounds, but the effective concentrations varied somewhat between strains (Table 1). Hygromycin B was the only drug that required heat shocking during development to provide sufficient drug resistance (Figure 1F). Blasticidin S was toxic in the resistant strain at high concentrations, and both counterselection agents, ganciclovir and 5-fluorocytosine, also showed general toxicity above a certain concentration (Table 1). Importantly, drug exposure is limited to a single generation during selection or counterselection for the desired genetic event, after which animal maintenance occurs on regular food. We never observed defects in marker-expressing strains, their fecundity, or overall health on regular or drug-treated food. Although there does remain the potential for mutagenicity, the widespread and continued use of these drugs in mammalian cell culture without reported genetic defects (Eglitis, 1991; Mortensen and Kingston, 2009) bodes well for their use in *Drosophila* and other organisms.

To evaluate whether different markers can be combined, we determined the specificity of marker-conferred drug resistance and sensitivity by testing each marker-expressing strain and the *EGFP* control on each of the functional drugs and found that all markers are drug specific (Figures 1I–1N).

### Multiplexed co-selection and/or co-counterselection produces genotypically pure populations

**Figure 2.**
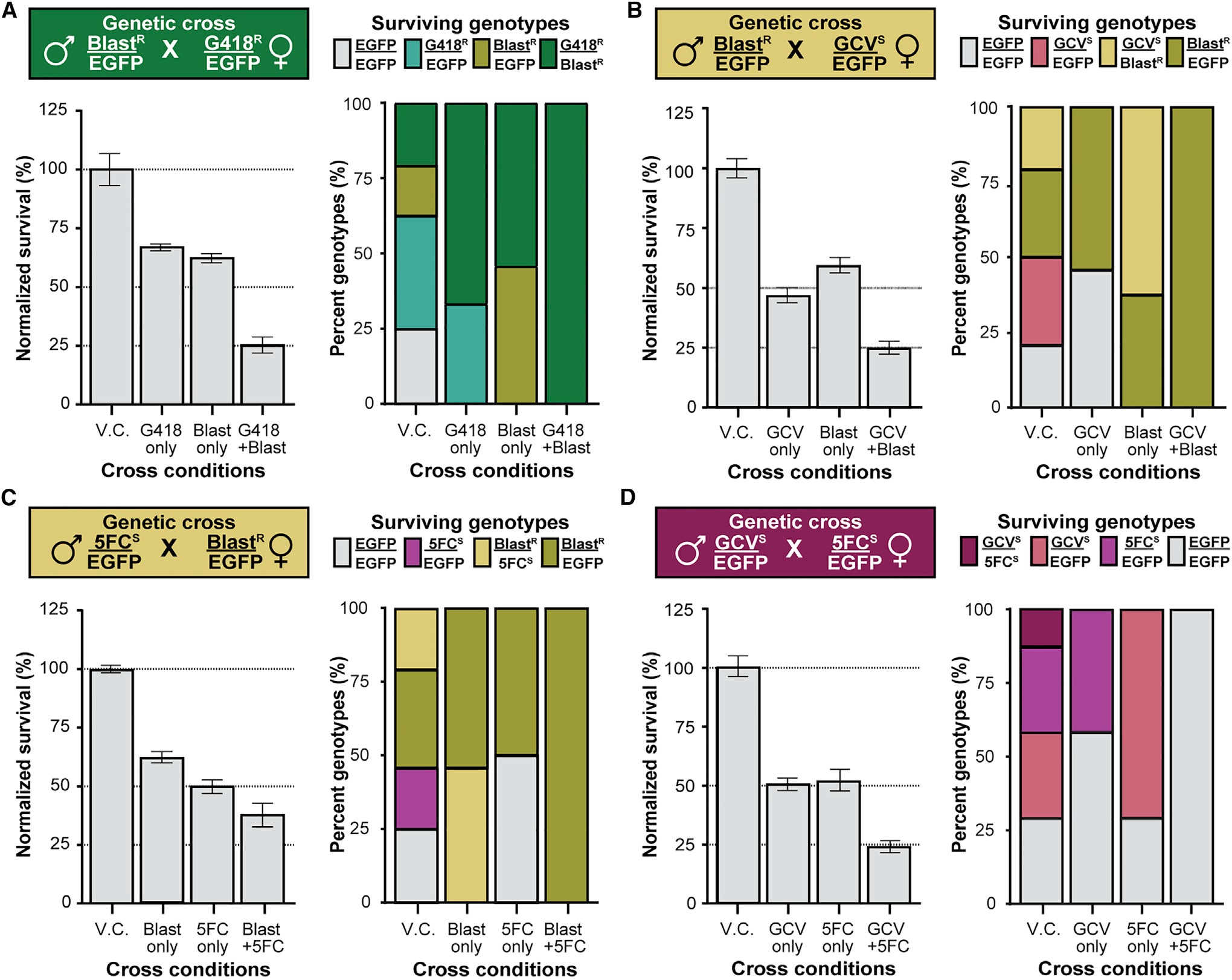
Multiplexed co-selection and/or co-counterselection produces genotypically pure populations Heterozygous drug-resistant and/or drug-sensitive fly strains: Blast^R^ and G418^R^ (A); Blast^R^ and GCV^S^ (B); 5FC^S^ and Blast^R^ (C); and GCV^S^ and 5FC^S^ (D) were crossed together under four drug conditions. Fly crosses were tested on food with vehicle control (VC), drug A, drug B, or both drugs (A+B). Survival data matched expected frequencies, with selection or counterselection reducing normalized percent survival by 50% and dual-drug treatment by 75% versus vehicle control. For each cross, 24 flies from each of the four drug conditions were collected and individually genotyped. Genotyping produced expected genotypes for each drug condition in each individual cross. Selection with a single drug results in survival of only the corresponding resistant genotypes (A–C). Conversely, single-drug counterselection eliminates only the relevant sensitized strains (B–D). Finally, dual-drug co-selection produced only the dually resistant genotype (A), only Blast^R^ heterozygotes survived combination selection and counterselection (A–C), and co-counterselection resulted in only EGFP homozygotes, sensitive to neither drug, surviving treatment (D). See also Figures S4–S6.

We next tested the robustness of combining multiple markers for co-selection, combination selection and counterselection, and co-counterselection (Figures 2A–2D, left, and S4). For each pair of marker heterozygotes, we set up crosses under four different drug conditions corresponding to the markers involved in the cross: vehicle control; drug 1; drug 2; and both drugs together. Each cross was set up twice, varying the sex of each marker heterozygote strain to account for potential parental effects on drug resistance or sensitivity (Figure S4). In each cross, there are four possible genotypes, whose proportions are genetically determined and modified by drug conditions (Figures 2A–2D, right, and S5).

Co-selection of the *G418^R^* and *Blast^R^* heterozygote cross in vials treated with vehicle control, G418 sulfate, blasticidin S, or both drugs at their respective ESCs produced the expected genotype frequencies in each drug condition (Figures 2A and S4A). Compared to vehicle control, normalized survival decreased by about 50% in the single-drug conditions and 75% when co-selected on both drugs, as expected. Genotyping confirmed that all four genotypes were represented in roughly equal proportion in control vials, whereas in single-drug-treated vials, only the expected resistant heterozygous (teal or olive bars) and dually resistant transheterozygote genotypes (green bars) survived drug treatment. In vials drugged with both G418 sulfate and blasticidin S, only the transheterozygous animals expressing both markers survived co-selection (green bars, Figure S5A). We observed similarly robust results for the other two selection marker cross combinations (Figures S4B, S4C, S5B, and S5C) and for the combination selection and counterselection crosses (Figures 2B, 2C, S4D, S4E, S5D, and S5E), as well as the co-counterselection crosses between *GCV^S^* and *5FC^S^* heterozygotes (Figures 2D, S4F, and S5F). In *Blast^R^* crosses where females conferred the blasticidin resistance marker, we found higher than expected survival frequencies, suggesting a maternal selection effect, as genotyping confirmed that all surviving progeny were *Blast^R^* positive (Figures S4 and S5).

Multiplexed drug-based selection and/or counterselection is analogous to the computer science concept of binary logic gates in which one or more inputs are computed into a single binary output using Boolean logic (Singh, 2014; Manzoni et al., 2016). Of the sixteen possible two-input binary Boolean logic gates, four commonly used gates can be directly represented using combinations of markers (AND, NOR, and both versions of NOT), whereas the other four commonly used gates (OR, NAND, XOR, and XNOR) can be generated indirectly by combining differentially selected and counterselected populations (Figure S6). Our experiments show that combining selection and/or counterselection drugs can generate a diverse set of genotypically pure populations in one or two generations, going beyond sex-specific selection of flies with a genetically engineered circuit coupled to G418 or puromycin resistance (Kandul et al., 2020). This combinatorial drug flexibility opens up opportunities for sophisticated genetic manipulations, crossing schemes, as well as population-control approaches.

### Single-step co-transgenesis via dual drug co-selection

**Figure 3.**
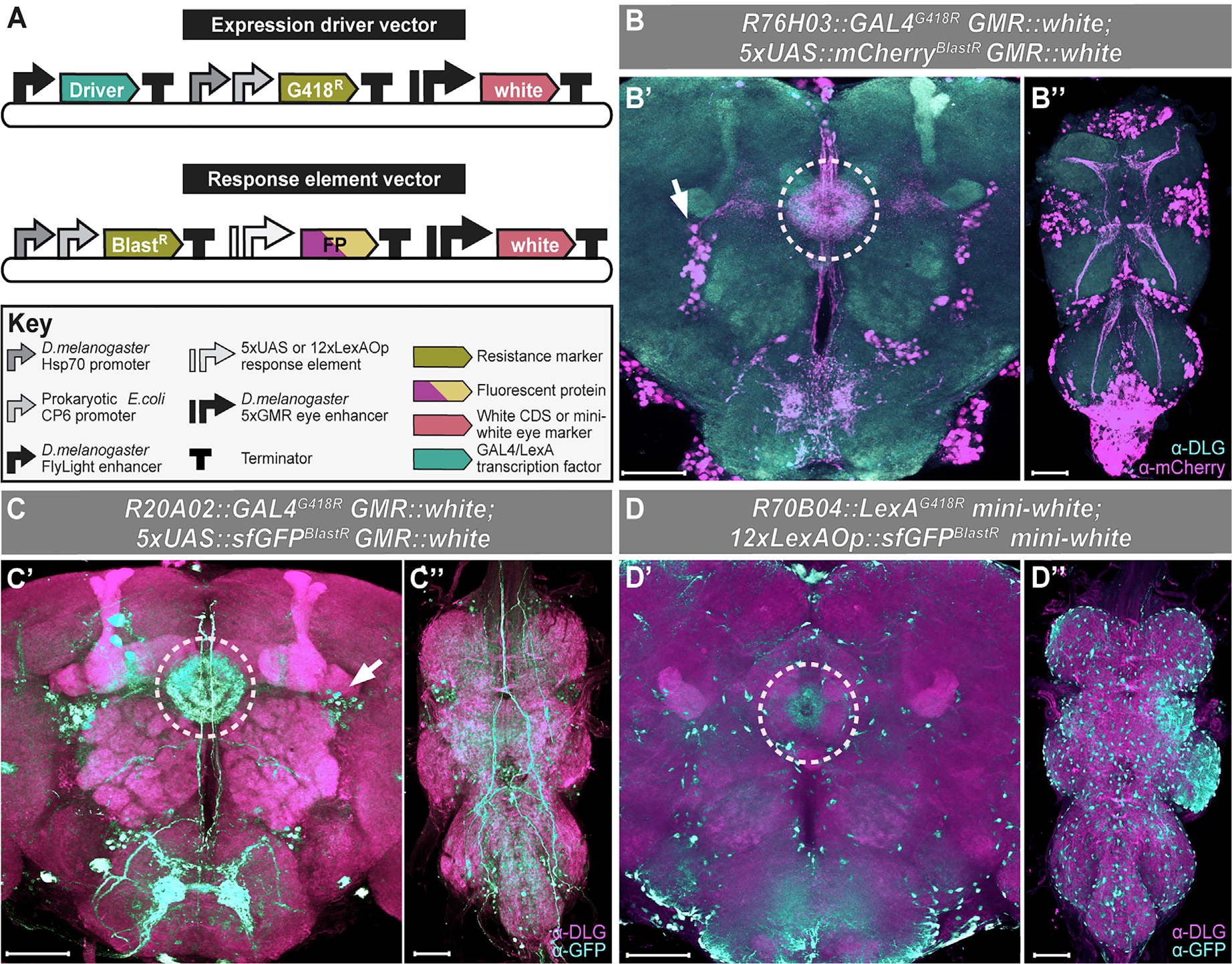
Single-step co-transgenesis via dual-drug co-selection (A) Schematic of the drug-selectable driver-response vector pairs for either the *GAL4/UAS* or the *LexA/LexAOp* binary expression systems. Driver vectors consist of a FlyLight library genomic enhancer element regulating *GAL4* or *LexA* transcription factor expression, a G418 resistance maker, and a visual eye marker. Response vectors contain a *5xUAS* or a *12xLexAOp* driver binding DNA motif upstream of a fluorescent protein (mCherry or *sfGFP*), a blasticidin resistance marker, and visual eye marker. Transgenics were generated via co-injections of driver-response vector pairs into a double-docking site fly line using ΦC31 integrase. (B–D) Results were visualized via immunofluorescent staining for the respective fluorescent proteins. (B) Staining for *R76H03::GAL4*-driven *mCherry* in the central complex of the adult fly brain showed expression in the ellipsoid body (dotted circle) and innervating R4 cells (arrow; B′), although in the ventral nerve cord, staining revealed an X-shaped pattern similar to FlyLight data (B″). (C) Staining for GFP in *R20A02::GAL4*; *UAS-GFP* adult fly brains labels the ellipsoid body (dashed circle) and R4 cells (arrow) in the brain, similar to previously reported expression of this enhancer (C′). Expression in the ventral nerve cord (VNC) shows less similarity to the previously reported pattern (C″). (D) Staining for *R70B04::LexA*-driven *sfGFP* showed only very faint expression in the ellipsoid body within the central complex compared to the described enhancer expression from FlyLight (D′), although in the VNC, expression was very similar to FlyLight expression for this enhancer (D″). Staining for Disc Large (*DLG*) was used as a counterstain. Scale bars represent 50 μm. See also Figure S7.

We next explored whether we could apply combinatorial drug selection to create genetically pure populations with direct practical significance. A simple but powerful demonstration of co-selection would be to make double-transgenic flies that carry both components of a binary expression system in a single-transgenic event. The original *GAL4/UAS* binary expression system and its later variants *LexA/LexAOp* and *QF/QUAS* have been powerful tools for functional analysis in *Drosophila* (Brand and Perrimon, 1993; Lai and Lee, 2006; Pfeiffer et al., 2010; Potter et al., 2010). However, generating such tools requires two separate transgenesis steps, each containing one half of the binary expression system (e.g., *GAL4* and *UAS*). The resulting transgenics must then be crossed together, resulting in a strain expressing both elements of the system (e.g., *GAL4*/*UAS*). Instead, we generated *GAL4/UAS* and *LexA/LexAOp* double-transgenic animals expressing the complete binary expression system in a single transgenesis step using dual drug co-selection. This eliminates both the need to screen transgenic progeny and the required crosses, simplifying generation of these tools. We first generated ΦC31 transgenesis-compatible *GAL4/LexA* driver and *UAS/LexAOp* response vectors. Driver vectors confer G418 resistance, and response vectors provide blasticidin S resistance (Figure 3A). We made different driver vectors under the control of enhancer elements from the FlyLight enhancer collection producing *pR76H03-GAL4^G418R^*, *pR20A02-GAL4^G418R^*, and *pR70B04-LexA^G418R^* (Jenett et al., 2012). The FlyLight collection is well characterized and therefore serves as a good control for the resulting expression patterns of our binary expression system vectors (Jenett et al., 2012). We cloned response vectors each composed of a synthetic *5xUAS* (Sarrion-Perdigones et al., 2013) or *12xLexAop* element (Table S1) driving expression of a fluorescent protein, either superfolder *GFP* (*sfGFP*) (Pédelacq et al., 2006) or monomeric Cherry (*mCherry*) (Shaner et al., 2004), resulting in vectors *p5xUAS-sfGFP^BlastR^*, *p5xUAS-mCherry^BlastR^*, and *p12xLexAOp-sfGFP^BlastR^* (Table S2). All driver and response vectors also contain a physical eye marker with a *5xGMR* enhancer element (Hay et al., 1994) driving expression of the *white* gene coding sequence or a synthetic *mini-white* eye marker (Figure 3A; Pirrotta, 1988). Driver-response vector pairs were injected into a double docking site (*attP*) fly strain (Table S3), and progeny were selected on G418 and blasticidin S dual-treated food. We successfully obtained double-transgenic flies for *pR76H03-GAL4^G418R^–p5xUAS-mCherry^BlastR^*, *pR20A02-GAL4^G418R^–p5xUAS-sfGFP^BlastR^*, and *pR70B04-LexA^G418R^*-*p12xLexAOp-sfGFP^BlastR^* driver-response element pairs.

The expression patterns in these strains are similar to the original FlyLight documented expression patterns (Figures 3B–3D; Jenett et al., 2012). Staining for the mCherry marker revealed expression in the central complex of adult brains of *R76H03::GAL4* animals marking the ellipsoid body and innervating R4 cells (Figure 3B′). Staining in the adult ventral nerve cord showed a similar X-shaped expression pattern as seen in the FlyLight collection (Figure 3B″). *R20A02*-driven *GAL4/UAS* binary expression labels the ellipsoid body and R4 cells in the adult brain similar to previously reported expression of this enhancer (Figure 3C′), although GFP expression in the ventral nerve cord (VNC) was less similar to the previously reported pattern (Figure 3C″). We observed only faint *sfGFP* expression in the ellipsoid body within the central complex in the adult brains of *R70B04::LexA*-driven *sfGFP* (Figure 3D′). Staining in the adult VNC showed a similar expression pattern as the equivalent *GAL4* enhancer line from the FlyLight collection (Figure 3D″). Differences between the observed expression patterns and those described in FlyLight are likely due to the use of different docking sites and differences in our cloned response elements affecting expression level. Despite these differences, our binary system reporter line shares significant similarities with previously reported expression patterns (Jenett et al., 2012). We also generated single *GAL4* driver transgenics for these enhancers and crossed them to a strong *UAS* reporter line (Pfeiffer et al., 2012). The resulting expression patterns were even more similar to those previously described, confirming that expression differences likely result from design and insertion site variations (Figures S7A–S7C; Jenett et al., 2012). Together, these experiments show that making double-transgenic flies in a single step could accelerate the generation of split-GAL4 reagents or orthogonal binary expression drivers and repressors among other alternative applications.

### Selection and counterselection to simplify balancer chromosome crosses

**Figure 4.**
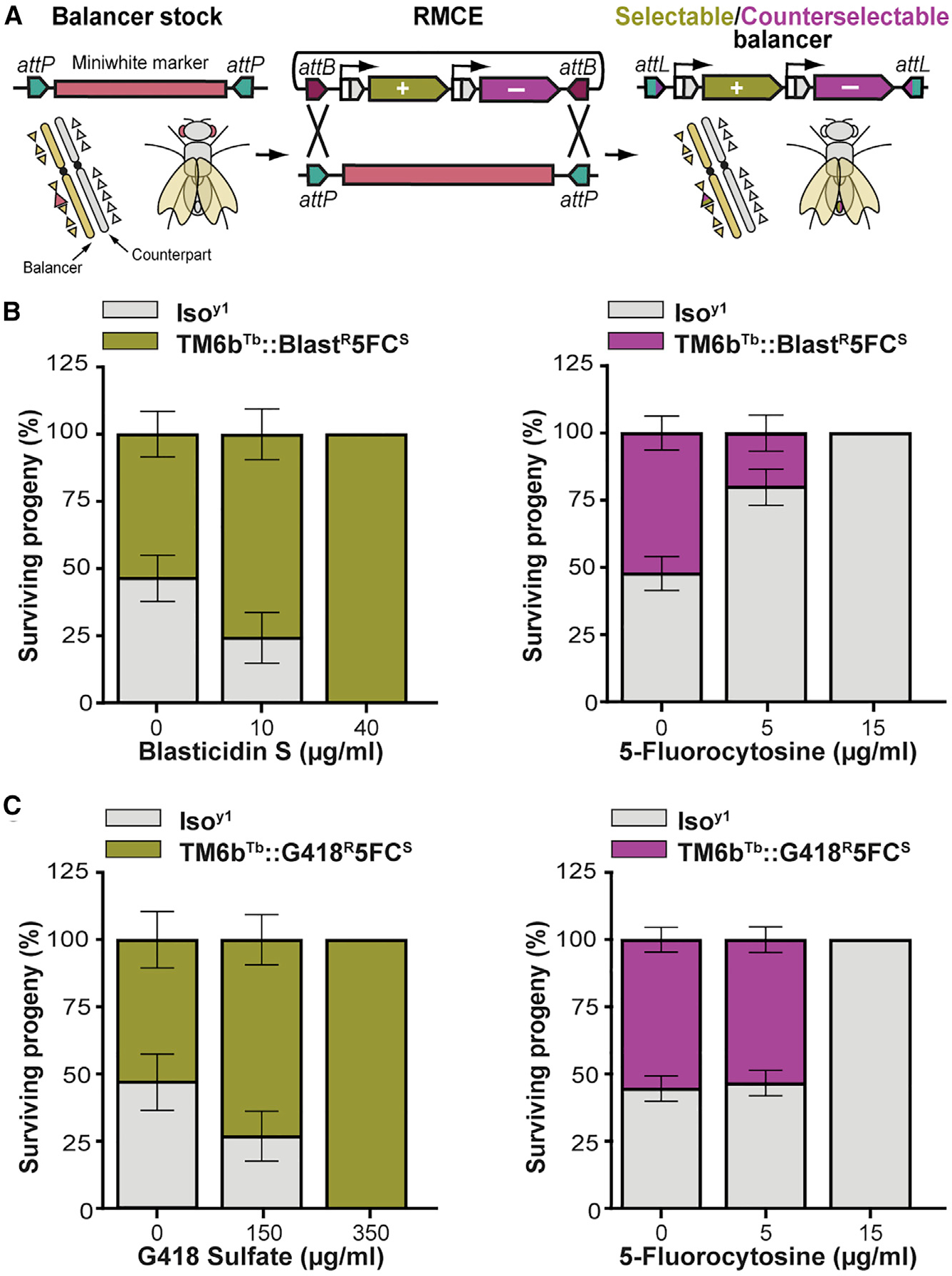
Selection and counterselection to simplify balancer chromosome crosses (A) Balancer chromosome fly stocks were generated via recombinase-mediated cassette exchange onto a *TM6B, Tb[1]* third chromosome balancer containing a double inverted *attP* docking site. (B and C) Two different versions of the balancer stock were generated resistant to blasticidin S or G418 with both sensitive to 5-fluorocytosine and abbreviated as *TM6b*^*Tb*^*::Blast*^*R*^*5FC*^*S*^ (B) and *TM6B*^*Tb*^*::G418*^*R*^*5FC*^*S*^ (C). An equal number of non-virgin *Iso*^*Y1*^ and balancer stock females, *TM6b*^*Tb*^*::Blast*^*R*^*5FC*^*S*^ (B) or *TM6B*^*Tb*^*::G418*^*R*^*5FC*^*S*^ (C), were mixed together in vials with varying concentrations of the appropriate drug. Populations selected with antibiotic produced only drug-resistant balancer animals at the respective drug ESCs, whereas concentration of 5-fluorocytosine had to be increased to 15 μg/mL for effective counterselection, likely due to lower basal marker expression in this docking site.

The ability to select for or against balancer chromosomes instead of screening and counterscreening them is useful when balancing transgenes or when only non-balancer offspring are needed for further analysis. Some dominant markers present on balancers or their dominantly marked counterparts are difficult to score, e.g., *Antp^Hu^* (*Humeral* [*Hu*]) on the third chromosome balancer *TM6B* (Miller et al., 2016) or *sna^Sco^* (*Scutoid* [*Sco*]) on the second balancer counterpart chromosome (Miller et al., 2018). Screening for the correct progeny in such crosses can be slow and tedious. Via recombinase-mediated cassette exchange (RMCE) (Bateman et al., 2006), we upgraded an existing third chromosome balancer (*TM6B* [*Tb*]), containing a *P* element insertion of a recombinase exchangeable cassette (Sun et al., 2012). We generated two selectable and counterselectable balancer chromosomes by combining either a blasticidin S or G418 resistance marker with a 5-fluorocytosine sensitivity marker expression cassette via synthetic assembly and then replacing the original *P* element insertion with our dual-marker cassette via RMCE (Figure 4A). The resulting transgenic strains have constitutive whole-body-marker expression that can be enhanced with heat shock treatment (Figures 4B and 4C).

We tested the selectable and counterselectable balancers by mixing equal numbers of non-virgin isogenized control (*Iso^Y1^*) and balancer strain females on vials with varying concentrations of the corresponding drugs. By exposing developing larvae to either blasticidin S (Figure 4B, left) or G418 (Figure 4C, left), we effectively selected for balancer flies. Conversely, we selected against the balancer strain via exposure to 5-fluorocytosine (Figures 4B and 4C, right). Basal marker expression was sufficient to confer G418 resistance, but heat shock treatment was required for complete blasticidin resistance, likely due to lower marker expression from this particular genomic locus (Figure 4B). The ECC for 5-fluorocytosine at 15 μg/mL was higher than in previous experiments, also likely due to position effects on marker expression. Heat shock induction would possibly reduce the amount of 5-fluorocytosine required for effective counterselection, though it is not required. A full set of selectable and counterselectable balancers as well as similar chromosomes for the Y and the 4^th^ chromosomes would also be useful to simplify some fly genetics experiments. Our experiments demonstrate the feasibility of such a resource.

### Drug-based selection of large fluorescently tagged *P[acman]* BAC transgenics to recapitulate endogenous expression patterns and identify protein complexes

**Figure 5.**
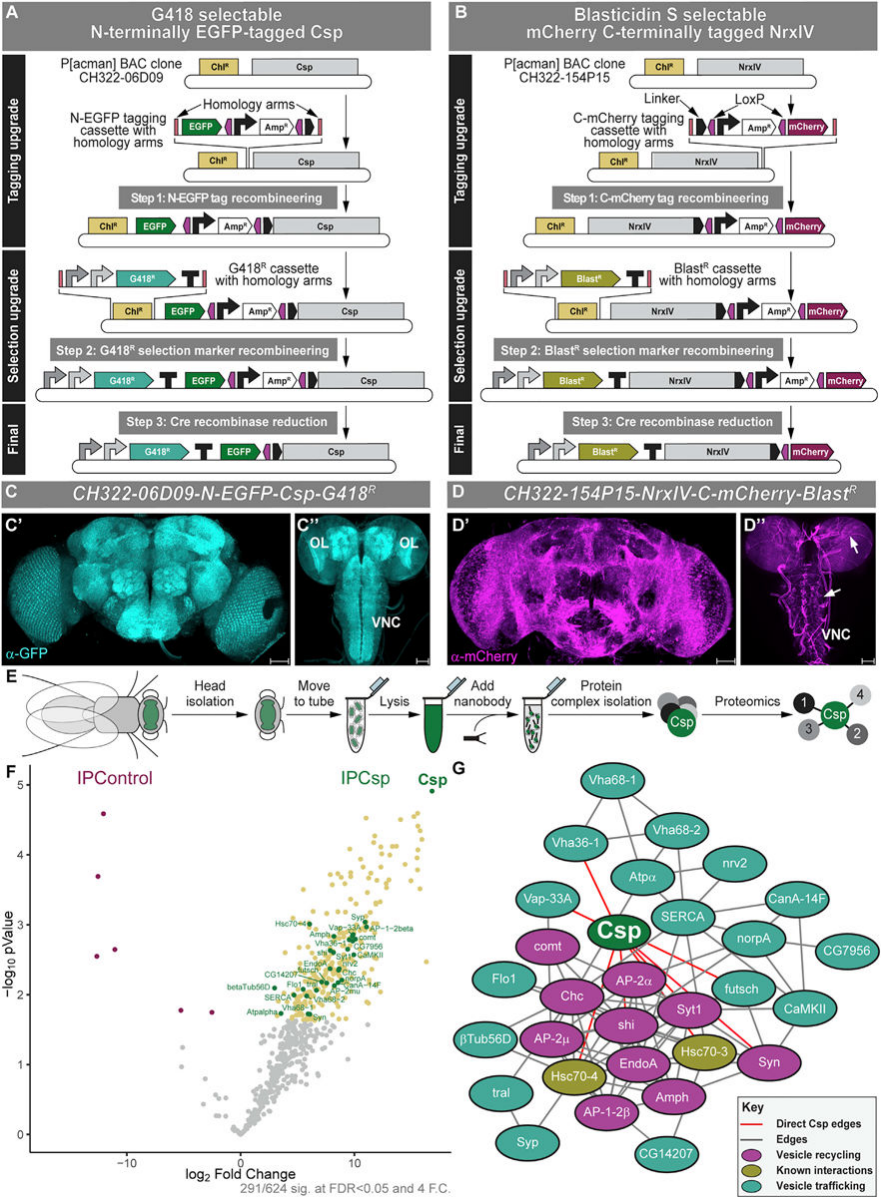
Drug-based selection of large fluorescently tagged *P[acman]* BAC transgenics to recapitulate endogenous expression patterns and identify protein complexes (A and B) Using a 3-step serial recombineering strategy, two genomic *P[acman]* clones were upgraded with a fluorescent tag, either on the N (A) or C terminus (B), and a selection marker expression cassette conferring resistance to G418 or blasticidin S. The bacterial ampicillin resistance marker (*Amp*^*R*^) was used to select successful recombineering reactions in the first step. This marker is removed via Cre-recombinase-mediated reduction in the third step. A short, flexible peptide sequence links the fluorescent tag in frame to the encoded gene (Figure S8). (C and D) Resulting selected flies were verified via immunofluorescent staining for tagged *P[acman]* BAC products. (C) Staining data for N-terminal *EGFP* tagged cystein string protein (Csp) shows strong neuropil expression throughout the adult fly brain (C′). In the larval brain, staining against tagged Csp shows clear neuropil expression in the visual system, including the optic lobes (OLs) as well as expression in the VNC (C″). (D) Staining for C terminus *mCherry*-tagged Neurexin IV (NrxIV) shows brain surface glial expression in the adult fly brain (D′). Similarly, staining shows glial expression in the larval brain with clear labeling of septate junctions formed at the borders of the surface subperineural glial (arrows) in the cerebral hemispheres and VNC (D″). Scale bars represent 50 μm. (E) Schematic of the workflow of the co-immunoprecipitation of GFP-tagged Csp from isolated fly heads followed by proteomic analysis. (F) Volcano plot shows differentially enriched proteins (by NCBI gene symbol) between tagged Csp and GFP control pull-downs. Colored dots represent peptides (291/624) that were found to be significantly different between the two groups at a false discovery rate (FDR) < 0.05 and enrichment of ≥4 fold. (G) Interactome analysis was performed with STRING. Shown is a selected interactome focusing on Csp and putative connections to vesicle recycling (purple) and other neuronal proteins (teal). Proteins in olive indicate known protein-protein interactions with Csp. See also Figures S8 and S9.

Large transgenes (>20 kb), such as *P[acman]* bacterial artificial chromosome (BAC) clones (Venken et al., 2006, 2009), are an important tool in *Drosophila* genetics, as they provide endogenous genomic context for genes of interest. They are the gold standard for null allele rescue experiments (Venken et al., 2006, 2009, 2010) and demonstration of expression and localization of a tagged gene product covered by the genomic clone (Venken et al., 2008, 2009). However, the large size of the genomic clones results in lower transgenesis efficiency, requiring more extensive screening compared to smaller transgenes (Venken et al., 2006, 2009, 2010). Drug-based selection eliminates the need to screen, providing a simplified method for isolating rare transgenic events. We generated two transgenic strains with drug selectable and fluorescently protein-tagged *P[acman]* BAC library genomic clones using a 3-step serial recombineering strategy for N-terminal (Figure 5A) and C-terminal tagging (Figure 5B; see also Figure S8). The *P[acman]* BAC clone *CH322–06D09* covering *cysteine string protein* (*Csp*) (38 kb) was upgraded with an N-terminal *EGFP* tag and a G418 resistance marker, although clone *CH322–154P15* covering *neurexin IV* (*NrxIV*) (>35 kb) was upgraded with a C-terminal *mCherry* tag and a blasticidin S resistance marker.

We stained adult fly brains for each tagged protein using antibodies against GFP (Figure 5C) or mCherry (Figure 5D). Consistent with the role of Csp in presynaptic vesicle exocytosis, we observed strong neuropil staining in the adult brain (Figure 4C′; Bronk et al., 2005). We also observed neuropil staining in the larval brain corresponding to previous reports (Figure 5C″; Eberle et al., 1998). Staining for mCherry-tagged NrxIV showed localization on the surface of the brain, corresponding to its known glial expression in the formation of septate junctions, in adult and larval brains (Figures 5D′ and 5C″; Baumgartner et al., 1996; Banerjee et al., 2008). In particular, staining in the larval brain clearly marks septate-junction-rich lateral borders of subperineural glia in the cerebral hemispheres and VNC (Figure 5D″; Carlson et al., 2000). These staining data show that we are able to modify BAC clones to be both selectable and fluorescently tagged.

We next assessed the applicability of our selection-based genetics platform to biologically relevant questions by performing immunoprecipitation followed by unbiased interactome analysis for one of our tagged-BAC clone transgenic strains. We chose to focus our analysis on N-terminally tagged Csp due to its role in synaptic vesicle exocytosis (Bronk et al., 2005). We pulled down EGFP-tagged Csp protein from fly heads and assessed the identities of co-immunoprecipitated proteins using mass spectrometry (Figure 5E). We identified 943 distinct peptides from our sample (Table S4), of which 624 could be 100% identified in either the tagged Csp or EGFP control groups. Of the 624 identified proteins, 291 exhibit significant differential enrichment between Csp and control samples at a false discovery rate (FDR) < 0.05 and fold enrichment of >4 versus control (Figure 5F). We found high enrichment of most proteins of both ribosomal subunits, making up 57 of the top 100 identified peptides by p value, suggesting whole ribosomes were pulled down (Figure S9). We also identified proteins associated with synaptic vesicle recycling or trafficking, resulting in a highly significant STRING network (Szklarczyk et al., 2019; Figure 5G). We detected Hsc70 peptides, which are known Csp interactors (Eberle et al., 1998; Tobaben et al., 2001; Nosková et al., 2011), and identified shibire and Comatose, whose human orthologs (*DNM1* and *NSF*, respectively) interact either physically or biochemically with the human Csp ortholog (*CSPα*; Zhang et al., 2012). We also identified histones and mitochondrial-associated peptides enriched in our tagged Csp sample versus control (Figure S9). Further experiments are necessary to confirm the biological significance of these interactions.

### A selection and counterselection vector library resource for iterative synthetic assembly

**Figure 6.**
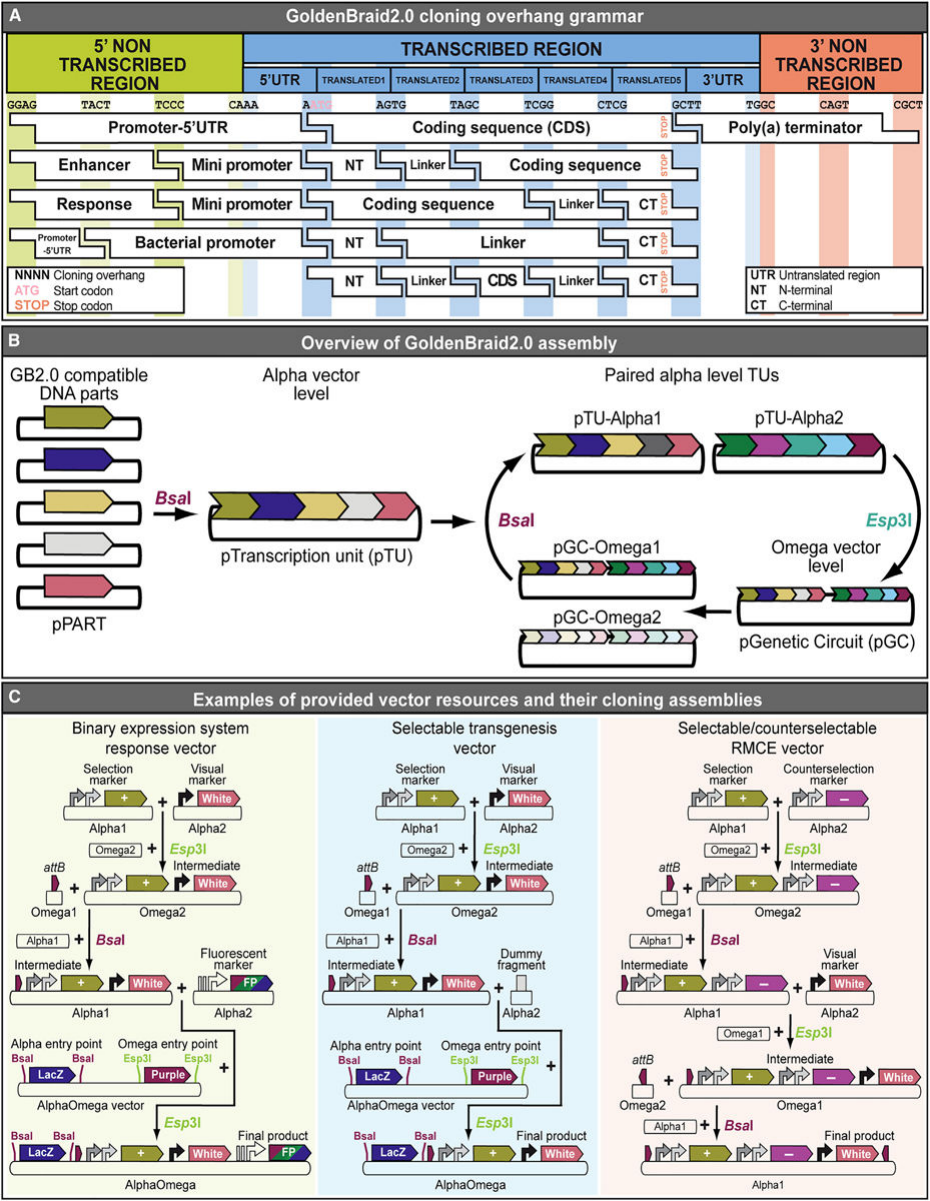
A selection and counterselection vector library resource for iterative synthetic assembly (A) Iterative synthetic assembly with Goldenbraid 2.0 (GB2.0) cloning uses a predefined “grammar” of four nucleotide overhangs that determine DNA element identity and assembly order during cloning. (B) GB2.0 uses a one-pot assembly of multiple DNA parts into one of two alpha-level destination vectors resulting in a transcription unit (TU). Paired TUs can be further assembled into an omega-level destination vector producing a multigenic genetic circuit (GC). These can then be further assembled back into an alpha-level vector to produce ever more complex constructs. (C) A number of pre-built selectable and/or counterselectable vectors are provided for a variety of applications as well as a library of GB2.0-compatible DNA elements totaling 121 plasmids. Each of these vectors was itself cloned from smaller elements using GB2.0 synthetic assembly. The vectors require minimal modification using either GB2.0 or traditional cut-and-paste cloning for a particular application. More experienced users can generate fully customized vectors appropriate to their needs using GB2.0 cloning. Three examples of vector resources and their cloning assemblies are shown. All plasmids are publicly available via Addgene (https://www.addgene.org/), and a full list can be found in Tables S1 and S2. See also Figure S10.

To make our selection and counterselection genetics platform easy to implement, we developed a vector library resource using GoldenBraid 2.0 cloning (GB2.0). GB2.0 is a multipartite, one-pot reaction synthetic assembly method that uses type IIs restriction enzymes, *Bsa*I and *Esp*3I (isoschizomer of *Bsm*BI), which cut away from their binding site to produce programmable four nucleotide overhangs (Engler et al., 2008; Sarrion-Perdigones et al., 2011, 2013). These overhangs produce a “grammar” that governs part assembly order in the reaction (Figure 6A). GB2.0 simplifies assembly of complex, multigenic constructs (Sarrion-Perdigones et al., 2013). Multiple compatible DNA elements can assemble in a single cloning reaction into a destination vector, either pColE1_Alpha1 or pColE1_Alpha2, via a *Bsa*I digest, resulting in a functional transcription unit plasmid (pTU) (Figures 6B, S10A, and S10B). GB2.0 is iterative, using the products of one assembly step as reagents in the next, allowing further assembly of paired pTUs into a second set of destination vectors, either pColE1_Omega1 or pColE1_Omega2, via an *Esp*3I reaction, resulting in a multigenic genetic circuit (GC) (Figure S10C; Sarrion-Perdigones et al., 2013). GB2.0 allows further assembly of paired GC products back into alpha destination vectors, the products of which are reagents for additional assembly steps in an iterative cloning process, resulting in ever more complex constructs (Figure 6B). GB2.0 construction schemes to generate complex constructs needed for co-selection (Figure 3) and combined selection and counterselection (Figure 4) demonstrate overall feasibility, although selectable *P[acman]* BAC transgenics (Figure 5) demonstrate the compatibility of our DNA library with existing DNA resources (Venken et al., 2009). Altogether, these examples illustrate the versatility and usefulness of this vector library resource for a broad range of potential applications (Figure 6C).

GB2.0 requires the use of compatible DNA elements with matching cloning overhangs and restrictions enzyme sites conforming to the established assembly grammar (Figure 6A; Sarrion-Perdigones et al., 2013). Here, we provide a collection of over 120 GB2.0-compatible vectors consisting of 64 DNA elements and 57 transgenesis ready vectors. Our DNA library consists of a variety of *Drosophila melanogaster* and non-*Drosophila* promoters, enhancers, markers (selection and counterselection, fluorescent, and physical), peptide linkers, tags, poly(A) terminators, and many other elements (Table S1). The provided toolkit of transgenesis-ready, drug-selectable and/or counterselectable vectors is compatible with GB2.0 and, in some cases, traditional restriction endonuclease cloning (Table S2). Included in the toolkit are (1) vectors for *in vivo* selection using one of four antibiotics (e.g., G418, puromycin, blasticidin S, and hygromycin B); (2) plasmids for generating drug-selectable *GAL4/UAS*, *LexA/LexAOp*, and *QF/QUAS* driver-response binary expression system vector pairs; (3) selectable and counterselectable RMCE-compatible vectors resistant to one of three drugs (G418, puromycin, or blasticidin S) and sensitive to 5-flurocytosine; and (4) selection and tagging cassettes for recombineering-based upgrading of genomic BAC clones (Table S2). The vector toolkit also contains all of the basic GB2.0 cloning vectors used in this work needed to adopt this cloning method. Because many of our vectors contain *attB* sites for ΦC31-integrase-mediated transgenesis, they can readily be transferred to additional insects, including other *Drosophila* and mosquito species (Holtzman et al., 2010; Labbé et al., 2010; Meredith et al., 2013; Pondeville et al., 2014; Volohonsky et al., 2015; Kudo et al., 2018), as well as vertebrates, such as fish and mice (Tasic et al., 2011; Kirchmaier et al., 2013; Mosimann et al., 2013; Roberts et al., 2014), that already have *attP* integration sites available.

## DISCUSSION

In this study, we developed a multiplex drug-based selection and counterselection strategy to simplify genetic manipulations in *Drosophila melanogaster*. Selection- and counterselection-based genetics eliminates the need to screen for or counterscreen against modified progeny via visual markers, significantly reducing workload. We generated four selection and two counterselection markers with broad applicability. Although previous efforts to adapt selection and counterselection genetics to *Drosophila* have had only limited success (Steller and Pirrotta, 1985; Handler and O’Brochta, 1991), our work demonstrates effective drug-based selection and counterselection to make genetically pure populations of animals through multiplexed marker co-selection, combination selection and counterselection, and co-counterselection. To demonstrate the power of multiplexing selection and counterselection drugs, we generated *GAL4/UAS* and *LexA/LexAop* double-transgenic animals in a single co-transgenesis step and made selectable and counterselectable balancer chromosomes. Generating transgenic fly lines for an experiment often takes 3 to 4 months to create and bring together multiple genetic elements into a single strain. Especially if using visible markers, this can involve time-consuming screening to identify progeny with the correct physical makers. Both applications eliminate the need for screening and complex cross schemes, making these genetic manipulations simpler and faster.

We also selected single transgenics for two different N- or C-terminally fluorescently tagged *P[acman]* BAC genomic library clones using either G418 or blasticidin S. We then analyzed the interactome of Csp by performing mass spectrometry on immunoprecipitated GFP-tagged Csp from fly heads and identified Hsc70–3, Hsc70–4, Shi, and Comt, known interactors of Csp or orthologs of interactors of human Cspα (Eberle et al., 1998; Nosková et al., 2011; Tobaben et al., 2001; Zhang et al., 2012). The co-immunoprecipitation of a large number of ribosomal subunit proteins suggests that Csp may also interact with the ribosome. Whether this interaction represents a role of Csp at the synapse or is due to aggregation because of modestly elevated Csp expression levels will require further investigation to validate its biological significance.

Choosing the right marker for a given application depends foremost on the site of insertion, as degree of resistance varies with marker expression. Although we designed our expression cassette to maximize marker expression, the genomic context of an insertion site does affect marker effectiveness. Expression can be enhanced with heat shock treatments but in our hands is not usually necessary. Our experience shows that the *nptII* marker is the most robust and should be the primary choice for most applications. The second preferred marker depends on application: for insertions into a well characterized, high expression docking site, the *bsr* marker (blasticidin S) is a good choice, but for insertions into a poorly expressing site, the *pac* marker is likely better, as puromycin does not seem to be toxic to resistant animals even at high concentrations (700 μg/mL), unlike blasticidin S. However, the high cost per vial for puromycin may be prohibitive for large-scale use. Finally, the *hph* marker is the least preferred marker, as heat shock is required for effective drug resistance.

Similar considerations apply to using multiple counterselectable drugs simultaneously. Although cost per vial is negligible for the counterselection markers, *sr39TK* or *FCU1*, degree of marker-conferred sensitivity also varies with insertion-site expression level. Importantly, for all counterselection drugs tested, there is a maximal concentration, beyond which even non-sensitized animals exhibit drug toxicity. The negligible cost of the counterselectable drugs makes their corresponding markers useful for RMCE-based (Bateman et al., 2006) upgrading of existing transposon insertions (e.g., MiMIC; Venken et al., 2011) or targeted CRISPR alleles (Zhang et al., 2014; Li-Kroeger et al., 2018; Kanca et al., 2019), after appropriately modifying them with a counterselectable marker.

We recommend testing innate drug resistance and sensitivity of a particular strain prior to using any of the markers (Matinyan et al., 2021), similar to establishing lethality curves in mammalian cell culture. Once established in a particular background, markers and their corresponding drugs can be used without further troubleshooting.

We modified a pair of balancer chromosomes by upgrading them with a selection and counterselection cassette. Although we only provide upgraded balancers for the third chromosome, multiple balancers exist for each of the three main *Drosophila* chromosomes and are often used in conjunction during complex crossing schemes. In the future, it may be possible to use multiple selectable balancers for different chromosomes.

Some small changes could further improve our drug-based genetics system. Substituting the *Hsp70* promoter for promoters from constitutively expressed genes, such as *α1-tubulin* (Angelichio et al., 1991; Zhang et al., 2013), *polyubiquitin* (Handler and Harrell, 2001), *actin 5C* (Angelichio et al., 1991), or *armadillo* (Vincent and Girdham, 1997), may provide higher and more consistent expression and may decrease variability between different genomic locations. Substituting the minimal HSV-TKpA transcriptional terminator (Steller and Pirrotta, 1985) for other polyadenylation signals, e.g., SV40 (Angelichio et al., 1991), may do the same. These modifications may improve the usefulness of the hygromycin B resistance marker especially.

To make our platform broadly useful, we generated a library of DNA elements and a transgenesis ready vector toolkit compatible with GB2.0 cloning for selection and counterselection genetics. This vector resource will make our next-generation transgenesis system immediately useful not just for the fly community but also for other model systems. The large library of DNA elements makes it possible for other labs to quickly build their own constructs and tools based on their needs. As all the markers are wholly exogenous to insects, selection and counterselection genetics can be readily applied to other drosophilids (Holtzman et al., 2010; Kudo et al., 2018) and other insect species like mosquitoes (Labbé et al., 2010; Meredith et al., 2013; Pondeville et al., 2014; Volohonsky et al., 2015; Matthews and Vosshall, 2020), provided careful selection of well-expressing promoters to ensure effective drug resistance and sensitivity. Obviously, the use of these markers in other species will have to be adapted to the particularities of their life cycles, life stages, and culture conditions. Robust selection and counterselection will likely be very valuable in species currently lacking available genetic tools and where transgenesis is not as efficient as in the fruit fly.

## STAR★METHODS

Detailed methods are provided in the online version of this paper and include the following:

### KEY RESOURCES TABLE

**Table:** 

REAGENT or RESOURCE	SOURCE	IDENTIFIER

Antibodies		

Polyclonal rabbit GFP	Invitrogen/Thermo Fisher	Cat# A11122, RRID:AB_221569
Mouse monoclonal anti-Dlg	Developmental Studies Hybridoma Bank	Cat# 4F3, RRID:AB_528203
Rat monoclonal mCherry	Invitrogen/Thermo Fisher	Cat# M11217, RRID:AB_2536611
Mouse monoclonal dCsp	Developmental Studies Hybridoma Bank	Cat# Ab49, RRID:AB_2307340
Chicken anti-rabbit AlexaFluor 488	Invitrogen/Thermo-Fisher	Cat# A-21441, RRID:AB_2535859
Goat anti-mouse AlexaFluor 568	Invitrogen/Thermo-Fisher	Cat# A11004, RRID:AB_2534072
Goat anti-rat AlexaFluor 568	Invitrogen/Thermo-Fisher	Cat# A-11077, RRID:AB_141874
Chicken anti-mouse AlexaFluor 568	Invitrogen/Thermo-Fisher	Cat# A21200, RRID:AB_2535786

Bacterial and virus strains		

Chemocompetent *Escherichia coli* strain DH10B	Invitrogen/Thermo Fisher Scientific	Cat# EC0113
Transformax *EPI300* electrocompetent strain cells	Epicenter/Lucigen	Cat# EC300110,
*EL350* recombineering strain bacterial cells	Kind gift from Donald Court, National Cancer Institute	n/a
*EC100D pir-116*	Epicenter/Lucigen	Cat# EC6P095H

Chemicals, peptides, and recombinant proteins		

G418 disulfate	VWR	Cat# 97063–060
Puromycin dihydrochloride	VWR	Cat# 97064–280
Blasticidin S hydrochloride	VWR	Cat# 71002–676
Hygromycin B	VWR	Cat# AAJ6068103
Ganciclovir	TCI America	Cat# G0315
5-Fluorocytosine	TCI America	Cat# F0321
*BsaI*-HFv2	New England Biolabs	Cat# R3733
*Esp*3I	New England Biolabs	Cat# R0734
*T4* DNA Ligase	Promega	Cat# M1801
iProof High-Fidelity DNA Polymerase	Biorad	Cat# 1725301
Phusion High-Fidelity DNA Polymerase	New England Biolabs	Cat# M0530
Q5 High-Fidelity DNA Polymerase	New England Biolabs	Cat# M0491
BioReady rTaq DNA polymerase	Bulldog Bio	Cat# BSAX050
Zeocin	Alfa Aesar	Cat# J67140
Phleomycin	VWR	Cat# AAJ67027–8EQ
*BbsI*-HF	New England Biolabs	Cat# R3539L
CopyControl Fosmid Autoinduction Solution	Epicenter/Lucigen	Cat# CCIS125
Trypsin protease	Gendepot	Cat# T9600
Ammonium bicarbonate	Fisher Scientific	Cat# 3003–1
LC-MS methanol	Fisher Scientific	Cat# A456
LC-MS water	J.T.Baker	Cat# JT9831–3
LC-MS acetonitrile	Fisher Scientific	Cat# A955
Formic Acid	Fisher Scientific	Cat# A117

Critical commercial assays		

QIAquick PCR Purification Kit	QIAGEN	Cat# 28106
QIAquick Gel Extraction Kit	QIAGEN	Cat# 28506
QIAprep Spin Miniprep Kit	QIAGEN	Cat# 27106
ChargeSwitch-Pro plasmid Miniprep kit	Invitrogen/Thermo Fisher Scientific	Cat# CS30250
ZR-96 Quick-gDNA kit	Zymo Research	Cat# D3010

Experimental models: Organisms/strains		

*Drosophila melanogaster*: EGFP: y[1]w[1118]; PBac{y[+mDint2] w[+mC] = P[acman]-attB-Hsp70-CP6-EGFP-TKpA} VK00033	This work, available from the BDSC	Cat# 92331, RRID:BDSC_92331
*Drosophila melanogaster*: G418^R^: y[1]w[1118]; PBac{y[+mDint2] w[+mC] = P[acman]-attB-Hsp70-CP6-nptII-TKpA} VK00033	This work, available from the BDSC	Cat# 92332, RRID:BDSC_92332
*Drosophila melanogaster*: Puro^R^:y[1]w[1118]; PBac{y[+mDint2] w[+mC] = P[acman]-attB-Hsp70-CP6-pac-TKpA} VK00033	This work, available from the BDSC	Cat# 92333, RRID:BDSC_92333
*Drosophila melanogaster*: Blast^R^: y[1]w[1118]; PBac{y[+mDint2] w[+mC] = P[acman]-attB-Hsp70-CP6-bsd-TKpA} VK00033/TM6B, Tb[1]	This work, available from the BDSC	Cat# 92334, RRID:BDSC_92334
*Drosophila melanogaster*: Hygro^R^: y[1]w[1118]; PBac{y[+mDint2] w[+mC] = P[acman]-attB-Hsp70-CP6-hph-TKpA} VK00033	This work, available from the BDSC	Cat# 92335, RRID:BDSC_92335
*Drosophila melanogaster*: Zeo^R^: y[1]w[1118]; PBac{y[+mDint2] w[+mC] = P[acman]-attB-Hsp70-CP6-bsr-TKpA} VK00033	This work, available from the BDSC	Cat# 92336, RRID:BDSC_92336
*Drosophila melanogaster*: GCV^S^:y[1]w[1118]; PBac{y[+mDint2] w[+mC] = P[acman]-attB-Hsp70-CP6-sr39TK-TKpA} VK00033	This work, available from the BDSC	Cat# 92337, RRID:BDSC_92337
*Drosophila melanogaster*: 5FC^S^: y[1]w[1118]; PBac{y[+mDint2] w[+mC] = P[acman]-attB-Hsp70-CP6-FCU1-TKpA} VK00033	This work, available from the BDSC	Cat# 92338, RRID:BDSC_92338
*Drosophila melanogaster*: FM7h-25C-1B: FM7h-RMCE-w[+mC]-25C-1B	This work, available from the BDSC	Cat# 92339, RRID:BDSC_92339
*Drosophila melanogaster*: CyO-25C-4A: y[1]w[1118]/Dp(1;y)y[+];CyO-RMCE-25C-4A(w[+mC])/L	This work, available from the BDSC	Cat# 92340, RRID:BDSC_92340
*Drosophila melanogaster*: CyO-52D-3A: y[1]w[1118]/Dp(1;y)y[+];CyO-RMCE-52D-3A(w[+mC])/L	This work, available from the BDSC	Cat# 92341, RRID:BDSC_92341
*Drosophila melanogaster*: TM6B-25C-5A: y[1]w[1118]/Dp(1;y)y[+];TM6b-RMCE-25C-5A(w[+mC])/D	This work, available from the BDSC	Cat# 92342, RRID:BDSC_92342
*Drosophila melanogaster*: TM6B-52D-1A: y[1]w[1118]/Dp(1;y)y[+];TM6b-RMCE-52D-1A(w[+mC])/D	This work, available from the BDSC	Cat# 92343, RRID:BDSC_92343
*Drosophila melanogaster*: *TM6b^Tb^::Blast^R^5FC^S^*: y[1]w[1118]/Dp(1;y)y[+];TM6b-RMCE{Blast^R^-5FC^S^-w[5xGMR-CDS]}-25C-5A(w[+mC])/D	This work, available from the BDSC	Cat# 92344, RRID:BDSC_92344
*Drosophila melanogaster*: *TM6b^Tb^*::*G418^R^5FC^S^*: y[1]w[1118]/Dp(1;y)y[+];TM6b-RMCE{G418^R^-5FC^S^-w[5xGMR-CDS]}-25C-5A(w[+mC])/D	This work, available from the BDSC	Cat# 92345, RRID:BDSC_92345
*Drosophila melanogaster*: 2xattP::VK00033;VK00020:y[1] M{RFP[3xP3.PB] GFP[E.3xP3] = vas-int.B}ZH-2A w[*]; PBac{y[+]-attP-3B}VK00033, PBac{y[+]-attP-9A}VK00020	This work, available from the BDSC	Cat# 92346, RRID:BDSC_92346
*Drosophila melanogaster*: N-EGFP-Csp: y[1]w[1118]; PBac{y[+mDint2] w[+mC] = P[acman]-attB-CH322-06D09-N-EGFP-Csp-G418}VK00033	This work, available from the BDSC	Cat# 92347, RRID:BDSC_92347
*Drosophila melanogaster*: NrxIV-CmCherry: y[1]w[1118]; PBac{y[+mDint2] w[+mC] = P[acman]-attB-CH322-154P15-NrxIV-C-Cherry-Blast}VK00033	This work, available from the BDSC	Cat# 92348, RRID:BDSC_92348
*Drosophila melanogaster*: R20A02::GAL4; w[5xGMR-CDS] = pR20A02-GAL4-G418^R^ } VK000XX, w[5xGMR-CDS] = p5xUASsfGFP-Blast^R^ }VK000XX 5xUAS::sfGFP:	This work, available from the BDSC	Cat# 92764, RRID:BDSC_92364
*Drosophila melanogaster*: R76H03::GAL4; 5xUAS::mCherry: w[5xGMR-CDS] = pR76H03-GAL4-G418^R^ }VK000XX, w[5xGMR-CDS] = p5xUAS-mCherry-Blast^R^ } VK000XX	This work, available from the BDSC	Cat# 92765, RRID:BDSC_92365
*Drosophila melanogaster*: R70B04::LexA; 12xLexAOp::sfGFP: w[+mC] = pR70B04-LexA-G418^R^ }VK000XX, w[+mC] = p12xLexAOp-sfGFP-Blast^R^ }VK000XX	This work, available from the BDSC	Cat# 92766, RRID:BDSC_92366
*Drosophila melanogaster*: *I*so^Y1^: y[1] w[67c23]	This work, available from the BDSC	Cat# 92349, RRID:BDSC_92349

Oligonucleotides		

TKpA-F	This work	N/A
TKpA-R	This work	N/A
attB-FOR	This work	N/A
attB-REV	This work	N/A
ModuleA-FOR	This work	N/A
ModuleA-REV	This work	N/A
ModuleF-FOR	This work	N/A
ModuleF-REV	This work	N/A
R6Kg-FOR	This work	N/A
R6Kg-REV	This work	N/A

Recombinant DNA		

p3xP3-Hsp70b	This work, available from AddGene	Cat# 165773
pMCS-TCCC	This work, available from AddGene	Cat# 165774
p5xGMR	This work, available from AddGene	Cat# 165775
pdSCP	This work, available from AddGene	Cat# 165776
pDmActin5c	This work, available from AddGene	Cat# 165777
p3xP3	This work, available from AddGene	Cat# 165779
pHsp70b-TACT	This work, available from AddGene	Cat# 165780
pDmAlfaTub84b	This work, available from AddGene	Cat# 165781
pDmVasa	This work, available from AddGene	Cat# 165783
pDmHsp70b	This work, available from AddGene	Cat# 165785
pDmPolyUbiq	This work, available from AddGene	Cat# 165786
pPromoterless-P2A	This work, available from AddGene	Cat# 165787
Promoterless TP10	This work, available from AddGene	Cat# 165790
pR20A02	This work, available from AddGene	Cat# 165791
pR70B04	This work, available from AddGene	Cat# 165792
pR76H03	This work, available from AddGene	Cat# 165793
pCP6-Dros	This work, available from AddGene	Cat# 165794
pCP6-STD	This work, available from AddGene	Cat# 165795
pCP7-STD	This work, available from AddGene	Cat# 165796
pTP10	This work, available from AddGene	Cat# 165797
pSV40L	This work, available from AddGene	Cat# 165798
pAdh Short 3′UTR	This work, available from AddGene	Cat# 165800
pHSV-TK	This work, available from AddGene	Cat# 165801
prrnBT1_STD	This work, available from AddGene	Cat# 165803
prrnBT2_STD	This work, available from AddGene	Cat# 165804
pL3S2P21_STD	This work, available from AddGene	Cat# 165805
pspy_STD	This work, available from AddGene	Cat# 165806
pGGGSx4	This work, available from AddGene	Cat# 165807
pT2A	This work, available from AddGene	Cat# 165809
pGGSx4 NT	This work, available from AddGene	Cat# 165810
pGGSx4 CT	This work, available from AddGene	Cat# 165811
p5xUAS-A1	This work, available from AddGene	Cat# 165812
4xLexAOp-A1	This work, available from AddGene	Cat# 165814
4xLexAOp-A2	This work, available from AddGene	Cat# 165815
4xLexAOp-O1	This work, available from AddGene	Cat# 165816
4xLexAOp-O2	This work, available from AddGene	Cat# 165817
5xQUAS	This work, available from AddGene	Cat# 165818
pGal4BD 1–147	This work, available from AddGene	Cat# 165819
pGAL4AD 768–881	This work, available from AddGene	Cat# 165820
pLexA BD	This work, available from AddGene	Cat# 165821
pVP16 AD	This work, available from AddGene	Cat# 165822
pQFBD	This work, available from AddGene	Cat# 165823
pQFAD	This work, available from AddGene	Cat# 165824
pGAL80	This work, available from AddGene	Cat# 165825
pQS	This work, available from AddGene	Cat# 165826
pmCherry	This work, available from AddGene	Cat# 165828
psfGFP	This work, available from AddGene	Cat# 165829
pEGFP	This work, available from AddGene	Cat# 165830
pEBFP2	This work, available from AddGene	Cat# 165831
pmCherry TL1	This work, available from AddGene	Cat# 165832
pmCherry TL5	This work, available from AddGene	Cat# 165833
pEGFP TL1	This work, available from AddGene	Cat# 165834
pEGFP TL5	This work, available from AddGene	Cat# 165835
pNPTII	This work, available from AddGene	Cat# 165836
pPAC	This work, available from AddGene	Cat# 165837
pBSR	This work, available from AddGene	Cat# 165838
pHPH	This work, available from AddGene	Cat# 165839
pBLE	This work, available from AddGene	Cat# 165840
pTK	This work, available from AddGene	Cat# 165841
pFCU1	This work, available from AddGene	Cat# 165842
pFC31 attB O1	This work, available from AddGene	Cat# 165843
pFC31 attB O2	This work, available from AddGene	Cat# 165844
pFC31	This work, available from AddGene	Cat# 165845
pWhite_CDS	This work, available from AddGene	Cat# 165846
pUPD2	This work, available from AddGene	Cat# 165856
pVD2_purple	This work, available from AddGene	Cat# 165857
P[acman]-A1	This work, available from AddGene	Cat# 165858
P[acman]-A2	This work, available from AddGene	Cat# 165859
P[acman]-O1	This work, available from AddGene	Cat# 165860
P[acman]-O2	This work, available from AddGene	Cat# 165861
pR6Kg-A1	This work, available from AddGene	Cat# 165862
pR6Kg-A2	This work, available from AddGene	Cat# 165863
pR6Kg-O1	This work, available from AddGene	Cat# 165864
pR6Kg-O2	This work, available from AddGene	Cat# 165865
pR6Kg-A1spm	This work, available from AddGene	Cat# 165866
pColE1-AlphaOmega	This work, available from AddGene	Cat# 165867
pEGFP-NT-Tagging	This work, available from AddGene	Cat# 165868
pEGFP-CT-Tagging	This work, available from AddGene	Cat# 165869
pmCherry-NT-Tagging	This work, available from AddGene	Cat# 165870
pmCherry-CT-Tagging	This work, available from AddGene	Cat# 165871
pMiniwhiteA1	This work, available from AddGene	Cat# 165872
pMiniWhiteA2	This work, available from AddGene	Cat# 165873
pGMR-WhiteA2	This work, available from AddGene	Cat# 165874
p3XP3-WhiteA2	This work, available from AddGene	Cat# 165875
pHSP70B-CP6-nptII	This work, available from AddGene	Cat# 165876
pHSP70B-CP6-pac	This work, available from AddGene	Cat# 165877
pHSP70B-CP6-bsr	This work, available from AddGene	Cat# 165878
pHSP70B-CP6-hph	This work, available from AddGene	Cat# 165879
pHSP70B-CP6-ble	This work, available from AddGene	Cat# 165880
pHSP70B-CP6-TK	This work, available from AddGene	Cat# 165881
pHSP70B-CP6-FCU1	This work, available from AddGene	Cat# 165882
pG418R-GMRWhite	This work, available from AddGene	Cat# 165883
pPuroR-GMRWhite	This work, available from AddGene	Cat# 165884
pBlastR-GMRWhite	This work, available from AddGene	Cat# 165885
pHygroR-GMRWhite	This work, available from AddGene	Cat# 165886
pRMCE{G418R-5FCS-GMRWhite}-VasaphiC31	This work, available from AddGene	Cat# 165887
pRMCE{PuroR-5FCS-GMRWhite}-VasaphiC31	This work, available from AddGene	Cat# 165888
pRMCE{BlastR-5FCS-GMRWhite}-VasaphiC31	This work, available from AddGene	Cat# 165889
pG418R-MCS-GAL4-MiniWhite	This work, available from AddGene	Cat# 165890
pPuroR-MCS-GAL4-MiniWhite	This work, available from AddGene	Cat# 165892
pBlastR-MCS-GAL4-MiniWhite	This work, available from AddGene	Cat# 165894
pG418R-MCS-LexA-VP16-GMRWhite	This work, available from AddGene	Cat# 165895
pG418R-MCS-LexA-VP16-MiniWhite	This work, available from AddGene	Cat# 165896
pPuroR-MCS-LexA-VP16-GMRWhite	This work, available from AddGene	Cat# 165897
pPuroR-MCS-LexA-VP16-MiniWhite	This work, available from AddGene	Cat# 165898
pBlastR-MCS-LexA-VP16-MiniWhite	This work, available from AddGene	Cat# 165900
pG418R-MCS-QF-MiniWhite	This work, available from AddGene	Cat# 165902
pBlastR-MCS-QF-MiniWhite	This work, available from AddGene	Cat# 165906
pBlastR-5xUAS-sfGFP-GMRWhite	This work, available from AddGene	Cat# 165907
pBlastR-5xUAS-mCherry-GMRWhite	This work, available from AddGene	Cat# 165908
pBlastR-5xUAS-sfGFP-MiniWhite	This work, available from AddGene	Cat# 165910
pBlastR-5xUAS-mCherry-MiniWhite	This work, available from AddGene	Cat# 165911
pBlastR-5xUAS-EBFP2-MiniWhite	This work, available from AddGene	Cat# 165912
pBlastR-12xLexAOp-sfGFP-MiniWhite	This work, available from AddGene	Cat# 165916
pBlastR-12xLexAOp-mCherry-MiniWhite	This work, available from AddGene	Cat# 165917
pBlastR-12xLexAOp-EBFP2-MiniWhite	This work, available from AddGene	Cat# 165918
pBlastR-5xQUAS-mCherry-GMRWhite	This work, available from AddGene	Cat# 165920
pBlastR-5xQUAS-EBFP2-GMRWhite	This work, available from AddGene	Cat# 165921
pBlastR-5xQUAS-sfGFP-MiniWhite	This work, available from AddGene	Cat# 165922
pBlastR-5xQUAS-mCherry-MiniWhite	This work, available from AddGene	Cat# 165923
pBlastR-5xQUAS-EBFP2-MiniWhite	This work, available from AddGene	Cat# 165924

Software and algorithms		

Prism 7 software v9.1.1	GraphPad Software	https://www.graphpad.com/scientific-software/prism/
Adobe Illustrator Creative Cloud	Adobe	https://www.adobe.com/creativecloud.html
Adobe Photoshop Creative Cloud	Adobe	https://www.adobe.com/creativecloud.html
Search Tool for the Retrieval of Interacting Genes/Proteins	STRING (https://www.string-db.org)	Szklarczyk et al., 2019
Cytoscape v3.8.2	https://cytoscape.org	Shannon et al., 2003
Zen Software Blue Version 2.3 pro HWL	Zeiss	https://www.zeiss.com/microscopy/us/products/microscope-software/zen.html
Codon Optimization Tool	IDT	http://www.idtdna.com/pages/tools/codon-optimization-tool?returnurl=%2FCodonOpt
Splice Site Prediction by Neural Network	Berkley Drosophila Genome Project	https://www.fruitfly.org/seq_tools/splice.html
SnapGene v4.2.3	GSL Biotech LLC	https://www.snapgene.com:443/products/snapgene/

Other		

ChromoTek GFP-Trap Dynabeads	ChromoTek	Cat# gtd-10, RRID:AB_2827592
NuPAGE 10%, Bis-Tris protein gel	Thermo Fisher Scientific	NP0315BOX
Reprosil-Pur Basic C18 beads	Dr.Maisch GmbH, Germany	R119.b9.3
Fused silica tubing	IDEX Health	FS-110

### RESOURCE AVAILABILITY

#### Lead contact

Further information and requests for resources and reagents should be directed to and will be fulfilled by the lead contact, Koen Venken; Email: koen.j.t.venken@gmail.com, Phone: 1-713-798-3698.

#### Materials availability

There are no restrictions on material availability of any reagent produced in this work. All plasmids generated in this study are made available through Addgene (http://www.addgene.org/). Generated plasmid materials and distinct identifiers are summarized in Tables S1 and S2 and the Key resources table. All fly stocks generated in this study (Table S3; Key resources table) are made available through the Bloomington *Drosophila* Stock Center (https://bdsc.indiana.edu/). Fly stocks, primer sequences used in this study and stock numbers are summarized in the Key resources table.

#### Data and code availability

Data for Figure 5F is available as Table S4. The mass spectrometry proteomics data have been deposited to the ProteomeXchange Consortium via the PRIDE partner repository with the dataset identifier PXD026579. This paper does not report original code. Any additional information required to reanalyze the data reported in this work paper is available from the Lead Contact upon request.

### EXPERIMENTAL MODEL AND SUBJECT DETAILS

#### Animals

Animals used in this study: *Drosophila melanogaster*, males and females, aged 2–10 days. *Drosophila melanogaster* strains used and developed in this study are listed in Table S3 and the Key resources table.

#### Bacteria

Microbial strains used in this study: Chemocompetent *Escherichia coli* strain DH10B (Invitrogen/Thermo Fisher Scientific), Transformax *EPI300* (Epicenter/Lucigen), strain *EL350* recombineering bacterial cells (kind gift from Donald Court, National Cancer Institute), and Transformax *EC100D pir-116* (Epicenter/Lucigen).

### METHOD DETAILS

#### Molecular biology enzymes

Restriction enzyme cloning was accomplished using: *Bsa*I-HFv2 (New England Biolabs, #R3733), Esp3I (New England Biolabs, R0734) and *T4* Ligase (Promega, M1801). PCR was performed using proofreading enzymes iProof High-Fidelity DNA Polymerase (Biorad, #1725301), Phusion High-Fidelity DNA Polymerase (New England Biolabs, M0530), or Q5 High-Fidelity DNA Polymerase (New England Biolabs, M0491). Molecular biology experiments, including *in silico* designs and experimentation, as well as plasmid maps, were designed using SnapGene software (https://www.snapgene.com:443/products/snapgene/) (GSL Biotech LLC). All plasmids developed in this project are listed in Tables S1 and S2 and the Key resources table.

#### General molecular biology

Molecular biology cloning was confirmed by agarose gel DNA electrophoresis after restriction enzyme digestion to expose diagnostic DNA bands of specific lengths, as well as control uncut plasmid to eliminate unwanted multimeric assemblies. All end products were verified by Sanger sequencing using GeneWiz (https://www.genewiz.com/) or Eurofins genomics (https://eurofinsgenomics.com/). PCR purifications, gel extractions and all plasmid preparations, excluding all bacterial artificial chromosome (BAC) backbone vectors, were performed using the QIAquick PCR Purification Kit (QIAGEN, #28106), QIAquick Gel Extraction Kit (QIAGEN, #28506), and QIAprep Spin Miniprep Kit (QIAGEN, #27106), respectively according to manufacturer’s instruction. All BAC plasmids were prepared using the ChargeSwitch-Pro plasmid miniprep kit (Invitrogen/Thermo Fisher Scientific, CS30250) according to manufacturer’s instruction. Primers were obtained from MilliporeSigma.

#### Chemical reagents

The following drugs were used: G418 sulfate (VWR 97063–060), puromycin HCl (VWR 97064–280), blasticidin S (VWR 71002–676), hygromycin B (VWR AAJ6068103), zeocin (Alfa Aesar J67140), phleomycin (VWR AAJ67027–8EQ), ganciclovir (TCI America 50–155-694), and 5-fluorocytosine (TCI America 50–014-34810).

#### Antibodies for immunofluorescence

The following primary antibodies were used for immunofluorescence: polyclonal rabbit GFP (Invitrogen/Thermo Fisher Cat# A11122, RRID:AB_221569, 1/500), mouse monoclonal anti-Dlg (Developmental Studies Hybridoma Bank Cat# 4F3, RRID:AB_528203, 1/100), rat monoclonal mCherry (Invitrogen/Thermo Fisher Cat# M11217, RRID:AB_2536611, 1/500), and mouse monoclonal dCsp (Developmental Studies Hybridoma Bank Cat# Ab49, RRID:AB_2307340, 1/100). Secondary antibodies were obtained from Invitrogen and were used at a final concentration of 1/500: chicken anti-rabbit AlexaFluor 488 (Invitrogen/Thermo-Fisher, Cat# A-21441, RRID:AB_2535859), goat anti-mouse AlexaFluor 568 (Invitrogen/Thermo-Fisher, Cat# A11004, RRID:AB_2534072), goat anti-Rat AlexaFluor 568 (Invitrogen/Thermo-Fisher, Cat# A-11077, RRID:AB_141874), and chicken anti-mouse AlexaFluor 568 (Invitrogen/Thermo-Fisher, Cat# A21200, RRID:AB_2535786). Images were captured with Zen Software (Blue Version 2.3 pro HWL, Zeiss).

#### Computational resources

All synthesized coding DNA sequences were first codon optimized for expression in *Drosophila melanogaster* using an online tool (http://www.idtdna.com/pages/tools/codon-optimization-tool?returnurl=%2FCodonOpt, IDT), and then analyzed for splice acceptor and donor sites using a splice site prediction online tool (https://www.fruitfly.org/seq_tools/splice.html, BDGP) which were then manually removed.

#### Dual-kingdom (counter)selection markers

We generated an expression cassette for the selection marker Neomycin phosphotransferase II/nptII (G418^R^) in the plasmid *pBluescript II KS (+)* (*pBS-KS*) (Stratagene/Agilent). The *Hsp70* promoter was amplified from plasmid template *pCasper-HS*, obtained from the *Drosophila* Genomics Resource Center (1215) (Steller and Pirrotta, 1986), using primers Hsp70-F and Hsp70-CP6-R. The *CP6* promoter was amplified from genomic DNA isolated from the *TP977* strain (kind gift from Anthony Poteete, University of Massachusetts Medical School) (Poteete et al., 2006) using primers Hsp70-CP6-F and CP6-Neo-R (for primers see Table S5 and the Key resources table). The open reading frame for a non-synthetic codon optimized version of G418 was amplified from plasmid template *pEGFP-N1* (Clontech/Takara Bio) using primers CP6-G418-F and HSVTK-R1. Next, a secondary overlap extension PCR (Horton et al., 1989) was performed using purified products of all three primary PCR products (*Hsp70* promoter, *CP6* promoter and G418^R^ open reading frame) as templates, and primers Hsp70-F and HSVTK-R2, resulting in the *Hsp70-CP6-G418^R^-HSV-TKpA* fragment. This fragment was cloned as a *Not*I cut fragment in a *Not*I linearized *pBS-KS* plasmid, resulting in *pBS-KS-Hsp70-CP6-G418-HSV-TKpA* maintained in the *DH10B* bacterial strain (Thermo Fisher Scientific) that can grow on LB plates with 100 μg/ml Ampicillin and 30 μg/ml Kanamycin, demonstrating functionality of the hybrid *Hsp70/CP6* promoter in bacteria.

A cloning cassette compatible with downstream sequence and ligation independent cloning (SLIC) (Li and Elledge, 2007), as well as chew-back, anneal and repair (CBAR) cloning, now commonly known as Gibson cloning (Gibson et al., 2009), was cloned in *attB-Pacman-ApR* (Venken et al., 2006). The Gibson cloning cassette consisting of the dual-kingdom promoter, *Hsp70-CP6*, separated from the *HSV-TKpA* transcriptional terminator, by a distinct *Nhe*I restriction enzyme site, was obtained by primary PCR amplification of the Hsp70-CP6 part using template *pBS-KS-Hsp70-CP6-G418-HSV-TKpA* and primers Hsp70-Ascl-F and Hsp70-CP6-TKpA·R1, followed by a secondary PCR using the primary template as PCR and primers Hsp70-Ascl-F and Hsp70CP6-TKpA-R2-Pacl, and cloned as an *Asc*I/*Pac*I cut fragment into an *Asc*I/*Pac*I linearized *attB-P[acman]-ApR* plasmid resulting in plasmid *P[acman]-attB-Hsp70CP6-NheI-TKpA*. Plasmid copy number induction was performed using fosmid autoinduction solution performed as described (Epicenter/Lucigen).

Selection/counterselection markers were commercially synthesized as *Drosophila* codon optimized fragments (Thermo Fisher Scientific) and amplified using SLIC and CBAR/Gibson compatible primers as follows: EGFP-SLIC was amplified from the synthetic construct encoding the *MiMIC* transposable element (Venken et al., 2011) with EGFP-F and EGFP-R; G418-SLIC was amplified from the G418 synthetic fragment (Davies and Smith, 1978) with G418-F and G418-R; Puro-SLIC was amplified from the Puro synthetic fragment (Vara et al., 1985) with Puro-F and Puro-R; Blast-SLIC was amplified from the Blast synthetic fragment (Itaya et al., 1990) with Blast-F and Blast-R; Hygro-SLIC was amplified from the Hygro synthetic fragment (Gritz and Davies, 1983) with Hygro-F and Hygro-R; Zeo-SLIC was amplified from the Zeo synthetic fragment (Oliva-Trastoy et al., 2005) with Zeo-F and Zeo-R; GCV-SLIC was amplified from the GCV synthetic fragment (Black, Kokoris and Sabo, 2001) with GCV-F and GCV-R; and 5FC-SLIC was amplified from the 5FC synthetic fragment (Erbs et al., 2000) with 5FC-F and 5FC-R. PCR amplified SLIC-fragments were subcloned in *Nhe*I linearized *F-2-5-attB-Hsp70-CP6-TKpA* as previously described (Li and Elledge, 2007), and annealing reactions transformed in home-made chemocompetent *EPI300* cells (Epicenter/Lucigen). Plasmid copy number induction was performed using fosmid autoinduction solution performed as described (Epicenter/Lucigen), resulting in plasmids: *P[acman]-attB-Hsp70-CP6-EGFP-TKpA*, *P[acman]-attB-Hsp70-CP6-G418-TKpA*, *P[acman]-attB-Hsp70-CP6-Puro-TKpA*, *P[acman]-attB-Hsp70-CP6-Blast-TKpA*, *P[acman]-attB-Hsp70-CP6-Hygro-TKpA*, *P[acman]-attB-Hsp70-CP6-Zeo-TKpA*, *P[acman]-attB-Hsp70-CP6-GCV-TKpA*, and *P[acman]-attB-Hsp70-CP6-5FC-TKpA*.

#### Transgenic selectable/counterselectable flies

Plasmids P[acman]-attB-Hsp70-CP6-EGFP-TKpA, P[acman]-attB-Hsp70-CP6-G418-TKpA, P[acman]-attB-Hsp70-CP6-Puro-TKpA, P[acman]-attB-Hsp70-CP6-Blast-TKpA, P[acman]-attB-Hsp70-CP6-Hygro-TKpA, P[acman]-attB-Hsp70-CP6-Zeo-TKpA, P[acman]-attB-Hsp70-CP6-GCV-TKpA, and P[acman]-attB-Hsp70-CP6-5FC-TKpA were prepared for microinjection using the QIAprep Spin Miniprep Kit (QIAGEN) according to the handbook and concentrations adjusted to 150 ng/μl. Transgenic flies were generated by microinjection using a custom injection stock, containing a germline driven ΦC31 source on the X (Bischof et al., 2007) (kind gift from Johannes Bischof and Konrad Basler, University of Zurich), and an attP docking site on the third chromosome (Venken et al., 2006): y[1] M{RFP[3xP3.PB] GFP[E.3xP3] = vas-int.B}ZH-2A w[*]; PBac{y[+]-attP-3B}VK00033 with putative integration events (identified by mini-white expression) balanced and correct integration events verified molecularly as previously described (Venken et al., 2009, 2010). The resulting strains are listed in Table S3 and the Key resources table.

#### Preparation of drug containing fly food

Fly food was made in a large kettle with mixer with the following ingredients per 1 l water: 6.4 g agar (Genesee Scientific, 66–103), 30 g yeast (Red Star, Webstaurant), 70 g cornmeal (Luby’s Cafeteria), 55 g dextrose (VWR, JT1910–5), 30 g sucrose (VWR, BDH9308), 4 mL 20% tegosept dissolved in Ethanol (Genesee Scientific, 20–258), and 4 mL propionic acid (Sigma-Aldrich, P1386). Agar is dissolved in heating water first, followed by adding yeast, cornmeal, dextrose and sucrose. When everything is dissolved, heat is turned down, fly food allowed to cool to 70°C, and tegosept and propionic acid carefully added to avoid bubbling.

A piston-driven food dispenser, DAB-8–4 (Filamatic) was used to precisely dispense 8 mL of food into each vial. Food was allowed to cool and air dry overnight in covered but unplugged vials. 20 holes were then poked three-fourths of the way down into the food using a home-made hole puncher. Appropriately solubilized drug was added to the fly food at 50X concentration, i.e., 160 μl for 8 ml. Drug was allowed to permeate into the food for 48 hours prior to introducing adult flies. Drugs were dissolved in MilliQ water (G418 sulfate, puromycin, blasticidin S, and hygromycin B), pH7.0 HEPES buffer (Zeocin and Phleomycin), 0.1N NaOH (ganciclovir) or 1x PBS (5-fluorocytosine).

#### Generation of the isogenized Iso^Y1^ strain

Fly strains with the following genotypes, *y[1] w[67c23]* (BDSC #6599) and *P{w[+mW.Scer\FRT.hs] = RS3}l(1)CB-6411-3[1], w[1118]/FM7h* (BDSC #6878), were used in conjunction with a fly stock double balanced for the second and third chromosomes, to generate a fly stock isogenized for all three major chromosomes: X (*y[1] w[67c23]* from BDSC #6599), 2 and 3 (both from BDSC #6878). This strain is abbreviated to *Iso^Y1^*.

#### Effective drug concentration determination

For each drug titration, self-crosses were set up between the relevant drug resistant or sensitive strain (*G418^R^*, *Puro^R^*, *Blast^R^*, *Hygr*o*^R^*, *Zeo^R^*, *GCV^S^*, or *5FC^S^*), or the control strain (*EGFP* or *Iso^Y1^*) with 3 mating pairs per vial to determine the effective selection/counterselection concentration (ESC or ECC). For each drug, four vials were set up per drug concentration per fly strain tested on said drug. Flies were allowed to mate and lay eggs for one week at 25°C in an acclimated incubator before being removed. Larvae were left to develop at 25°C for an additional two weeks. Surviving adult flies in each vial at the end of three weeks, if any, were counted, and results normalized to vehicle control treated vials and reported as percent survival ± SEM. We averaged at least three replicates per drug concentration per drug as occasionally a vial would not result in a fertile cross. Heat shock induction of marker expression was performed as follows: crosses with 3 pairs per vial were setup on vials with drug and allowed to lay overnight at 25°C. After 24 hours, developing eggs and larvae were heat shocked by placing vials into a 37°C water bath for 30 min. Care was taken to ensure vials were only 3/4^th^ submerged in water to improve adult fly survival while still providing heat shock to eggs and larvae in the food. After 30 min, vials were removed, dried, and placed back into the incubator. Heat shock was repeated every 2 days for a week before adults were discarded and remaining larvae allowed to develop at 25°C for an additional 2 weeks as above. This frequency balances marker expression with overall survival of heat shock treatment. Significance of change in percent survival per strain per drug concentration was determined via 2-way ANOVA using Dunnett’s multiple comparisons test at α = 0.05. A detailed protocol for determining effective drug concentrations is provided elsewhere (Matinyan et al., 2021).

#### Specificity of marker/drug combinations

Drug specificity for each selection and counterselection marker was characterized by exposing all marker expressing strains, except *Zeo^R^* (*G418^R^*, *Puro^R^*, *Blast^R^*, *Hygr*o*^R^*, *GCV*^S^, or *5FC*^S^) as well as the control strain (EGFP) to each of the six drugs (G418, puromycin, blasticidin S, hygromycin B, ganciclovir, or 5-fluorocytosine) at their determined ESC or ECC (Figures 1C–1H). Strain self-crosses were setup as described above with four crosses per strain on food containing drug or vehicle control, added as described above. Results were averaged with at least three replicates per strain per drug and normalized to vehicle control treated vials and reported as percent survival. Statistical significance was determined via multiple t test using the Holms-Sidack method with an α = 0.05.

#### Robustness of marker multiplexing

Homozygous drug resistant and drug sensitive fly strains were crossed to homozygous control (*EGFP*) flies to generate marker/EGFP heterozygotes: *G418^R^/EGFP*, *Puro^R^/EGFP*, *Blast^R^/EGFP*, *GCV^S^/EGFP* and *5FC^S^/EGFP*. Heterozygous fly strains were then crossed to each other as follows: *G418^R^/EGFP* to *Blast^R^/EGFP*, *Puro^R^/EGFP* to *G418^R^/EGFP*, *Blast^R^/EGFP* to *Puro*^R^*/EGFP*, *Blast^R^/EGFP* to *GCV^S^/EGFP*, *Blast^R^/EGFP* to *5FC^S^/EGFP*, and *5FC^S^/EGFP* to *GCV^S^/EGFP*. All six cross schemes were set up with reciprocal male and female parents. Each cross was further subdivided into four crosses under different drug conditions: vehicle control, one of the drugs, the other drug, or both drugs. Food was drugged by adding either 160 μl of a single drug at 50X its ESC/ECC or 80 μl of both drugs at 100X ESC/ECC when two drugs were used. Vehicle control vials were made with 160 μl of vehicle control if both drugs shared the same solvent or with 80 μl of each different solvent if the two drugs were in different solvents. Fly crosses were setup as described above with four replicates per drug conditions per experiment. The number of surviving adult flies was normalized to vehicle control in each experiment and results were reported as percent survival averaged from at least three fertile replicates per cross condition per experiment. On occasion one of four crosses per condition would not produce progeny and was excluded from analysis.

#### Genotyping dually (counter)selected flies

From each of the crosses described above, 24 flies were collected randomly for each of the four drug conditions. Single flies were put into each well of a 96-well plate along with a 5 mm stainless steel ball and 500 μL of genomic lysis buffer of the ZR-96 Quick-gDNA kit (Zymo Research, D3010). Plates were sealed and placed into a tissue homogenizer, 1600 MiniG (SPEX SamplePrep). Flies were homogenized for 45 s at 1000 rpm. DNA was extracted using the ZR-96 Quick-gDNA kit according to the manufacturer’s instructions (Zymo Research). Flies were genotyped using a set of three PCRs per extracted DNA plate each using BioReady rTaq DNA polymerase (Bulldog Bio, BSAX050) and primer pairs specific to one of the three potential markers (Table S5; Key resources table).

#### Generation of a double-docking site fly stock

A double attP docking site chromosome was generated by crossing y[1] M{RFP[3xP3.PB] GFP[E.3xP3] = vas-int.B}ZH-2A w[*]; PBac{y[+]-attP-3B}VK00033 males (Venken et al., 2009, 2010) to y[1] M{RFP[3xP3.PB] GFP[E.3xP3] = vas-int.B}ZH-2A w[*]; PBac{y[+]-attP-9A}VK00020 females (Venken et al., 2009, 2010), resulting in transheterozygous female progeny with the genotype, y[1] M{RFP[3xP3.PB] GFP[E.3xP3] = vas-int.B}ZH-2A w[*]; PBac{y[+]-attP-3B}VK00033/PBac{y[+]-attP-9A}VK00020 that were backcrossed to double balanced males, w[*] dlg1[14] P{w[+mW.hs] = FRT(w[hs])}101/FM7a; PBac{w[+mC] = PB}CG11583[c01124] P{ry[+t7.2] = neoFRT}80B/TM3, Sb[1] (BDSC #36283). Single double balanced flies, containing y[1] M{RFP[3xP3.PB] GFP[E.3xP3] = vas-int.B}ZH-2A w[*] and putatively a recombinant double attP docking site chromosome, PBac{y[+]-attP-3B}VK00033, PBac{y[+]-attP-9A}VK00020, were backcrossed to double balanced flies, whose double balanced progeny was self-crossed and homozygosed, generating the y[1] M{RFP[3xP3.PB] GFP[E.3xP3] = vas-int.B}ZH-2A w[*]; PBac{y[+]-attP-3B}VK00033, PBac{y[+]-attP-9A}VK00020 stock. Presence of both docking sites was confirmed by PCR amplification from genomic DNA from putative 2x docking site stain flies using primers VK33_RIGHT_F, VK33_RIGHT_R, VK33_LEFT_F, and VK33_LEFT_R for VK00033 and VK20_RIGHT_F, VK20_RIGHT_R, VK20_LEFT_F and VK20_LEFT_R for VK00020 (see Table S5 and the Key resources table for primer sequences). Flies are available from the Bloomington *Drosophila* Stock Center (https://bdsc.indiana.edu/; see Table S3 and the Key resources table).

#### GoldenBraid 2.0 cloning reactions

GoldenBraid 2.0 cloning (GB2.0) was used to generate and assemble all DNA parts and vector toolkit elements. GB2.0 cloning is an assembly method where all part vectors, destination vector, restriction enzyme, and ligase are mixed together in a single reaction (Sarrion-Perdigones et al., 2011, 2013). Briefly, 40 ng of destination vector and 40 ng of each donor-part vector are mixed with 1 μL of the appropriate restriction enzyme (*Bsa*I-HFv2 or *Esp*3I, New England BioLabs), 1 μL of *T4* Ligase (Promega), and 2 μL of the Ligase 10x Buffer (Promega) in a final volume of 20 μL. Reactions were set up in a standard thermocycler (Applied Biosystems) with at least 25 cycles of digestion/ligation reactions (2′ at 37°C, 5′ at 16°C with a final extension step of 10’ at 72°C). More cycles are recommended for complex assemblies of more than 3 parts or involving parts with large size differences (> 2kb) up to 50 cycles.

GB2.0 cloning depends on an extensive library of compatible DNA elements as reagents for assembly steps (Sarrion-Perdigones et al., 2011, 2013). DNA parts are incorporated into the system, or domesticated, by addition of appropriate GB2.0 cloning overhangs (the grammar of which determines the order of part assembly) via PCR amplification or commercial synthesis of compatible fragments (Figure S10A; Engler et al., 2008; Sarrion-Perdigones et al., 2013). Fragments are then cloned into one of three universal part domesticator (*pUPD*) vector backbone via *Esp*3I enzyme digestion: *pUPD* (kind gift from Diego Orsaez, The Institute for Plant Molecular and Cellular Biology), *pUPD2* (this work), and *pUPD3* (Addgene, #118043) (Sarrion-Perdigones et al., 2019), followed by transformation of 2 μl of reaction product into chemocompetent *E.coli DH10B* cells (Epicenter/Lucigen; Sarrion-Perdigones et al., 2013, 2019). All domesticated parts were confirmed via restriction endonuclease fingerprinting (New England BioLabs), agarose gel electrophoresis visualization and sequence verified by Sanger sequencing (GeneWiz, Eurofins Genomics). GB2.0 vectors and parts construction are described below.

#### Cloning of selectable GAL4/UAS vector pairs

*GAL4/UAS* and *LexA/LexAop* binary systems driver and response vectors were cloned using GB2.0. *GAL4* or *LexA* driver vector assembly began with cloning of a G418 resistance cassette, as described above, with one of three *GAL4*- or *LexA*-expressing cassettes, each driven by distinct DNA enhancer elements, previously characterized by the FlyLight consortium to produce intermediates *pR76H03-GAL4^G418R^*, *pR20A02-GAL4^G418R^*, and *pR70B04-LexA^G418R^* (Jenett et al., 2012). Expression cassettes consist of GB2.0 compatible enhancer element fragments, R76H03, R20A02, or R70B04 (Jenett et al., 2012) were assembled with the *Drosophila* synthetic core promoter *dSCP* fragment (Pfeiffer et al., 2008), *GAL4* or *LexA* DNA binding domain fragment, *GAL4^BD1−147^* or *LexA^BD^*, (Luan et al., 2006) flexible peptide linker fragment, GGGSx4 (4 repeats of gly-gly-gly-ser; Venken et al., 2011), *GAL4* (*GAL4^AD768−881^*) or *VP16* (*VP16^AD^*) activation domain fragment (Luan et al., 2006), and the late transcription terminator of simian virus 40, *SV40L* (Angelichio et al., 1991). Intermediates were then further assembled with a ΦC31 *attB* site fragment (Venken et al., 2006) as described above, to produce intermediates *pattB-R76H03-GAL4^G418R^, pattB-R20A02-GAL4^G418R^*, *pattB-R70B04-GAL4^G418R^*, and pattB-R70B04-LexA^G418R^. A final assembly with a visible eye marker cassette, as described above, produced the final driver vectors, *pattB-R76H03-GAL4^G418R^-5xGMRwhite*, *pattB-R20A02-GAL4^G418R^-5xGMRwhite*, *pattB-R70B04-GAL4^G418R^-5xGMRwhite*, and *pattB-R70B04-LexA^G418R^-Mini-white* (Table S2; Key resources table). *UAS/LexAop* response vectors began by assembly of a blasticidin S resistance cassette, as described above, with one of three *UAS* reporter cassettes consisting of a synthetic *5xUAS* or a 4xLexAOp element (Sarrion-Perdigones et al., 2013), *dSCP* part (Pfeiffer et al., 2008), *Drosophila* codon optimized coding sequence parts for one of two fluorescent proteins, *sfGFP* (Pédelacq et al., 2006) or *mCherry* (Shaner et al., 2004), and *SV40L* (Angelichio et al., 1991). Assembly produced intermediates *pUAS-sfGFP^BlastR^*, *pUAS-mCherry^BlastR^*, and *pLexAOp-sfGFP^BlastR^*. These were then further assembled with a ΦC31 *attB* site (Groth et al., 2004) to produce *pattB-UAS-sfGFP^BlastR^*, *pattB-UAS-mCherry^BlastR^*, *pattB-LexAOp-sfGFP^BlastR^*. Final assembly with a visible eye marker cassette, as described above, produced the final *UAS/LexAop* response vectors, *pattB-UAS-sfGFP^BlastR^-GMRwhite*, *pattB-UAS-mCherry^BlastR^-GMRwhite*, *pattB-LexAOp-sfGFP^BlastR^-Miniwhite* (Table S2; Key resources table).

#### Single-step, dual selection co-transgenesis

Plasmids pairs pR76H03-GAL4^G418R^-5xGMRwhite and pUAS-mCherry^BlastR^-5xGMRwhite, pR20A02-GAL4^G418R^-5xGMRwhite and pUAS-sfGFP^BlastR^-5xGMRwhite, and pR70B04-LexA^G418R^–Mini-white and pLexAOp-sfGFP^BlastR^–Mini-white were mixed together in a 1:1 ratio by weight (ng) at a final concentration of ~500 ng/ul, and co-injected into early stage embryos of the y[1] M{RFP[3xP3.PB] GFP[E.3xP3] = vas-int.B}ZH-2A w[*]; PBac{y[+]-attP-3B}VK00033, PBac{y[+]-attP-9A}VK00020 double attP docking site fly strain, abbreviated J15; VK00033, VK00020. Surviving adults were individually crossed to Iso^Y1^ flies of opposite sex on food with G418 sulfate (300 μg/ml) and Blasticidin S (35 μg/ml) as previously described. Transgenic offspring were then individually balanced with a third chromosome balancer line (w^1118^; Sb/TM6b) on regular food. Resulting balanced progeny were then self-crossed to generate third chromosome isogenized, double transgenic GAL4/UAS or LexA/LexAop stocks. Flies are available through the Bloomington *Drosophila* Stock Center (https://bdsc.indiana.edu/; Table S3; Key resources table).

#### Testing synthetic GAL4 driver functionality

Prior to generating dual transgenic binary expression system animals, we first tested the expression and function of our generated *GAL4* driver vectors. Transgenic animals were generated as described above and selected for and visually confirmed by screening for expression of 5xGMR *white*, producing a strong red eye phenotype, in selected animals. Transgenic animals were then crossed to a previously described strong *UAS* reporter line *P{10XUAS-IVS-GFP}attP2* (BDSC #32201) (Pfeiffer et al., 2010). Adult brains from the resulting progeny were dissected and immunostained for *GFP* expression as described below (Figure S7).

#### Immunofluorescent staining

Staining and imaging was performed as previously described (Gnerer et al., 2015). Briefly, adult brains were dissected in ice-cold PBS and fixed in 4% PFA/PBS for one hour. Next, the brains were rinsed 3 times with PBS-0.5% Triton X-100 (PBT) and then washed three times for 20 minutes in PBT at room temperature. The brains were then blocked in 5% normal goat serum (NGS) in PBT for one hour at room temperature. Samples were incubated in 5% NGS/PBT with primary antibody for 48 hours at 4°C. After three short rinses and three 20 minute washes in PBT, brains were incubated in 5% NGS/PBT with secondary antibody for 48 hours at 4°C. Brains were then rinsed three times and washed three times for 20 minutes at room temperature and then for 2 days at 4°C. Finally, brains were mounted in SlowFade mounting medium (Invitrogen/Thermo Fisher Scientific) and covered with a no. 0 glass coverslip that was separated from the slide by two strips of scotch tape. Immunostained brains were imaged with an upright Zeiss fluorescence Microscope (Axio Imager M2) equipped with an ApoTome2 (Zeiss) and a Hamamatsu Flash 4.0 V3 sCMOS Camera (Hamamtsu Photonics).

#### Generation of upgradeable balancer stocks

Double *attP* docking site-containing balancer chromosomes for the 1^st^, 2^nd^, and 3^rd^ chromosomes, *FM7c*, *CyO*, and *TM6B, Tb[1]*, respectively, were generated through *P* element mobilization using the *P* transposase source Δ2–3 (Robertson et al., 1988), obtained from the Bloomington *Drosophila* Stock Center, *y[1] w[1]; Ki[1] P{ry[+t7.2] = Delta2-3}99B* (BDSC #4368), and available double *attP* docking sites previously generated for ΦC31 integrase-mediated cassette exchange, located on the second chromosome (25C and 52D) (kind gifs of Jack Bateman, Bowdoin College; Bateman et al., 2006). *P* element-mediated mobilization resulted in five double *attP* docking site-containing balancer chromosomes that are available from the Bloomington *Drosophila* Stock Center (https://bdsc.indiana.edu/), abbreviated to *FM7c-25C-1B*, *CyO-25C-4A*, *CyO-52D-3A*, *TM6B-25C-5A*, *TM6B-52D-1A*, respectively (see Table S3 and the Key resources table).

#### Selection & counterselection RMCE cassettes

GB2.0 cloning was used to generate three all-in-one recombinase mediated cassette exchange (RMCE) genetic circuits. Each vector consists of one of three drug resistance markers (conferring resistance to G418, blasticidin S, or puromycin) and the 5-fluorocytosine sensitivity marker along with a synthetic *white* coding sequence visual eye marker all flanked on either end by inverted *attP* sites. All vectors also express a germline ΦC31 integrase source (Bischof et al., 2007) in the backbone. Briefly, each of the three resistance marker cassettes was combined with the 5-fluorocytosine sensitivity cassette, resulting in cloning intermediates *pG418^R^-5FC^S^*, *pBlast^R^-5FC^S^* or *pPuro^R^5FC^S^*. Marker cassettes are GB2.0 compatible versions of our dual-kingdom expression cassette generated via GB2.0 assembly from basic DNA parts. Next, a ΦC31 *attB* site (Groth et al., 2004) fragment was added onto the 5′ end of each intermediate to generate *pattB-G418^R^-5FC^S^*, *pattB-Blast^R^-5FC^S^ or pattB-Puro^R^5FC^S^*. These were then each assembled with a synthetic visual eye marker cassette to produce *pattB-G418^R^-5FC^S^-5xGMR*white, *pattB-Blast^R^-5FC^S^-5xGMR*white or *pattB-PuroR5FC^S^-5xGMRwhite*. A second inverted ΦC31 *attB* site (Groth et al., 2004) fragment was cloned onto the 3′ end of each intermediate to produce *pattB-G418^R^-5FC^S^-5xGMRwhite-attB*, *pattB-Blast^R^-5FC^S^-5xGMRwhite-attB* or *pattB-PuroR5FC^S^-5xGMRwhite-attB*. These were then cloned together with a germline expressing ΦC31 integrase cassette (Groth et al., 2004; Bischof et al., 2007) to generate the final all-in-one RMCE vectors: *pRMCE{G418^R^-5FC^S^}*, *pRMCE{Blast^R^-5FC^S^}*, and *pRMCE{Puro^R^-5FC^S^}* (Table S2; Key resources table).

#### Selection/counterselection balancer stocks

Transgenic flies were generated by microinjection of plasmids *pRMCE{G418^R^-5FC^S^}* and *pRMCE{Blast^R^-5FC^S^}* into a *TM6B, Tb[1]* RMCE balancer stock. Surviving adult flies were crossed to two *Iso^y1^* strain animals of the opposite sex on untreated food as previously described. Transgenic progeny were screened based on the visual marker described above, then backcrossed to the original balancer strain and finally self-crossed. Stocks are available through the Bloomington *Drosophila* Stock Center (https://bdsc.indiana.edu/; Table S3; Key resources table).

#### Validating selection/counterselection stocks

Balancer chromosome stocks were validated by mixing equal numbers of non-virgin balancer females and non-virgin *Iso^Y1^* stock females in vials drugged with varying concentrations of G418, blasticidin S, or 5-fluorocytosine up to the previously determined ESC and ECC for *Iso^Y1^*. Initial ECC experiments were repeated using higher 5-fluorocytosine concentrations as the original ECC was not fully effective at eliminating all balancer strain flies. Four replicates of two *Iso^Y1^* and two balancer chromosome stock females were set up per drug concentration per drug per balancer chromosome as described above. Surviving animals were visually sorted by genotype and counted. Results were reported as the average percentage of surviving flies of each genotype.

#### Selection/tagging cassettes for BAC upgrading

GB2.0 compatible G418 and blasticidin dual-kingdom marker expression cassettes were PCR amplified using overhang primers Marker-RECO1-F and Marker-RECO1-R to add 50 base pair arms on both 5′ and 3′ ends with homology to regions flanking the chloramphenicol bacterial resistance marker common to *P[acman]* BAC library genomic clones. Cassettes were then further amplified by secondary PCR using primers Marker-RECO2-F and Marker-RECO2-R to add GB2.0 cloning and *Xba*I restriction enzyme sites on both 5′ and 3′ ends (Table S5; Key resources table). Both cassettes were then cloned using GB2.0 into a compatible conditionally replicative vector backbone (Rakowski and Filutowicz, 2013), *pGB2-R6Kγ-A1^Spm^*, and sequence verified via commercial Sanger sequencing, resulting in plasmids, *pG418^R^-RECO* and *pBlast^R^-RECO* (Figure S8A). Similarly, a pair of fluorescent tag cassettes expressing either *EGFP* (Cormack et al., 1996) for N-terminal or *mCherry* (Shaner et al., 2004) for C-terminal protein tagging was cloned using GB2.0 methodology into an Alpha level GB2.0 vector as previously described (Sarrion-Perdigones et al., 2013). Cassettes consist of a human *EF1a* promoter piece (Kim et al., 1990) (cloning purposes only), an *EGFP* (Cormack et al., 1996) or *mCherry* coding sequence (Shaner et al., 2004), a *loxP* flanked ampicillin bacterial resistance marker, and an N- or C-terminal *GGGSx4* peptide linker (Venken et al., 2011). Tagging cassettes were then PCR amplified using primers, Csp-RECO1-F and Csp-RECO1-R for the N-terminal *EGFP* cassette, and Nrx-RECO1-F and Nrx-RECO1-R for the C-terminal *mCherry* cassette, adding the 50 base pair of homologous sequence required for recombineering. In a secondary PCR, GB2.0 cloning and *Xho*I restriction cut sites were added using primers Csp-RECO2-F and Csp-RECO2-R for the N-terminal variant, and Nrx-RECO2-F and Nrx-RECO2-R for the C-terminal cassette. Both PCR fragments were then GB2.0 cloned into vector *pGB2-R6Kγ-A1^Spm^* and Sanger sequence verified resulting in plasmids *pEGFP^NT^-RECO* and *pmCherry^CT^-RECO* (Figure S8B).

#### Serial recombineering of P[acman] BAC clones

Genomic *P[acman]* BAC clones *CH322–06D09* (Cysteine String Protein *Csp*), and *CH322–154P15* (Neurexin IV protein, *NrxIV*) (Venken et al., 2009) were isolated as described above and electroporated into strain *EL350* recombineering bacterial cells (kind gift from Donald Court, National Cancer Institute; Lee et al., 2001; Venken et al., 2009). The selection marker and tagging cassettes described above were linearized from their respective *pGB2-R6Kγ-A1^Spm^* vector backbones via *Xba*I or *Xho*I digestion, respectively. The genomic *P[acman]* BAC clones were upgraded through serial recombineering, using *Xho*I-released tagging cassettes during a first recombineering step, *Xba*I-released selection cassettes during a second recombineering step, and finally Cre-recombinase mediated reduction of the ampicillin resistance marker as previously described (Figure S8D; Venken et al., 2008). The resulting modified clones, *CH322-06D09-N-EGFP-Csp-G418* and *CH322-154P15-NrxIV-C-Cherry-Blast* were isolated from *EL350* cells, electroporated into, electrocompetent *EPI300* cells (Epicenter/Lucigen), and DNA was isolated as previously described (Venken et al., 2006, 2009, 2010).

#### Selectable/tagged P[acman] BAC transgenics

Upgraded P[acman] BACs CH322-06D09-N-EGFP-Csp-G418, and CH322-154P15-NrxIV-C-Cherry-Blast were microinjected singly into a dual ΦC31 integrase source, single docking site fly stock, y[1] M{RFP[3xP3.PB] GFP[E.3xP3] = vas-int.B}ZH-2A w[*] v[1] P{y[+t7.7] = nos-ϕ31\int.NLS}X; PBac{y[+]-attP-3B}VK00033 (BDSC #32543) as previously described (Venken et al., 2009, 2010). Surviving adults were then crossed to Iso^y1^ flies on food with either G418 sulfate (350 μg/ml) or Blasticidin S (35 μg/ml) as previously described. Putative integration events were balanced as previously described via crossing to a third chromosome balancer strain (w^1118^; Sb/TM6b) on un-drugged food (Venken et al., 2006, 2009). Balanced transgenics were then isogenized via self-cross on untreated food to generate the final homozygous transgenic lines. Integration events were verified molecularly as previously described (Venken et al., 2009). The resulting flies are available through the Bloomington *Drosophila* Stock Center (https://bdsc.indiana.edu/; Table S3; Key resources table).

#### Co-immunoprecipitation of eGFP-tagged Csp

Equal number of adult male and female flies were frozen in liquid nitrogen. Approximately 4,500 fly heads were collected by sieving the frozen flies. The heads were homogenized in chilled NETN buffer (50mM Tris pH 7.3, 170mM NaCl, 1mM EDTA, 0.5% NP-40, and protease and phosphatase inhibitors) using a 1600 MiniG automated tissue homogenizer and cell lyser (SPEX SamplePrep, NJ, USA) at 1250 rpm for 20 s in 6 rounds with 30 s gaps between each round when samples were on ice. The lysates were centrifuged at 12,000 g for 5 minutes to pellet the exoskeleton and undissolved material. The lysates were then sonicated and ultracentrifuged at 100,000 g for 20 minutes at 4°C to clear any undissolved material, cellular polymers, or insoluble lipids. The clear supernatant from this step was used for the immunoprecipitation. The lysates were incubated with ChemoTek GFP-Trap Dynabeads (ChromoTek, Cat# gtd-10, RRID:AB_2827592) previously equilibrated with ice cold NETN buffer. Following the 1 hour of incubation period the beads were washed with ice cold NETN buffer. To elute the proteins, beads were boiled in 2X SDS-PAGE sample loading buffer and subjected to SDS-PAGE. The gels were analyzed by Coomassie staining prior to mass spectrometry.

#### Mass spectrometry analysis of Csp interactome

The immuno-precipitated samples were resolved on NuPAGE 10% Bis-Tris Gel (Life Technologies) and the gel pieces were processed for in-gel digestion using trypsin enzyme (Gendepot T9600). The tryptic peptides were analyzed on nano-LC 1000 system (Thermo Fisher Scientific, San Jose, CA) coupled to an Orbitrap Fusion (Thermo Fisher Scientific, San Jose, CA) mass spectrometer. The peptides were loaded on a two-column setup using a pre-column trap of 2cm × 100μm size (Reprosil-Pur Basic C18 1.9 μm, Dr. Maisch GmbH, Germany) and a 20cm × 75μm analytical column (Reprosil-Pur Basic C18 1.9 μm, Dr.Maisch GmbH, Germany). The peptides were resolved on a 110-minute gradient of 6%–30% acetonitrile/0.1% formic acid at a flow rate of 200nl/min. The eluted peptides were directly electro-sprayed into the mass spectrometer operated in the data-dependent acquisition (DDA) mode. The full MS scan was acquired in Orbitrap in the range of 300–1400 m/z at 120,000 resolution, followed by Top35 MS2 in an ion trap (HCD 30% collision energy), with 5 s dynamic exclusion time. The MS/MS spectra were searched using the Mascot algorithm (Mascot 2.4, Matrix Science) against the *Drosophila melanogaster* NCBI refseq protein database (01/14/2019 download) in the Proteome Discoverer (PD 2.1, Thermo Fisher) interface. The precursor mass tolerance was confined to 20 ppm, fragment mass tolerance of 0.5 Da, and a maximum of two missed cleavages was allowed. Dynamic modification of oxidation on methionine, protein N-terminal Acetylation and DeStreak on cysteine was allowed. The gene product inference and iBAQ-based label free quantification was performed by gpGrouper algorithm (Saltzman et al., 2018). The mass spectrometry proteomics data have been deposited to the ProteomeXchange Consortium (Deutsch et al., 2020) via the PRIDE partner repository (Perez-Riverol et al., 2019) with the dataset identifier PXD026579.

#### Basic GoldenBraid 2.0 cloning vectors

We generated several basic GB2.0 cloning vectors with different backbone functionalities. Vectors were cloned using GB2.0 assembly via *Bbs*I-HF (New England Biolabs, R3539L) mediated cloning reaction as previously described (Sarrion-Perdigones et al., 2013). Briefly, vector backbone elements were either commercially synthesized as GB2.0 cloning compatible fragments or PCR amplified from existing plasmids with GB2.0 compatible cloning overhangs (Figure 6A). The *pUPD2* DNA element domestication vector was cloned by first synthesizing a *Lac*Z expressing GB2.0 entry cassette (Sarrion-Perdigones et al., 2013) flanked on either side by pairs of inverted bacterial transcription terminator elements, rrnBT1 (Green et al., 1985), rrnBT2 (Green et al., 1985), L3S2P21 (Chen et al., 2013), and spy (Clarke et al., 2018), as described above with *Bsa*I restriction sites on both 5′ and 3′ ends of the fragment. The synthesized element was cloned into a universal part domesticator vector, *pVD2_purple*, via *Bsa*I cloning reaction described below. The *pVD2_purple* vector features a purple chromophore screening marker and is itself derived from a previously described *pVD2* domesticator vector (Sarrion-Perdigones et al., 2013). All other backbone elements of *pUPD2* were PCR amplified as a single *Bbs*I restriction site flanked amplicon using primers pUPD_FOR and pUPD_REV using the *pUPD* vector as template (Sarrion-Perdigones et al., 2013). The amplified backbone elements were assembled and circularized with *pUPD2* entry cassette via *Bbs*I cloning reaction described below. The resultant vector was transformed into chemocompetent *DH10B* cells (Invitrogen/Thermo Fisher Scientific) and correct assemblies were identified using standard blue-white colony screening. Plasmid DNA was prepared using the QIAprep Spin Miniprep Kit (QIAGEN) and characterized via restriction enzyme digestion. Results were visualized via gel electrophoresis on 1% agarose gel. Correct clones were fully Sanger sequenced via commercial service (GeneWiz).

GB2.0 compatible *P[acman]* vectors were assembled by first amplifying backbone elements using the original *P[acman]* empty backbone vector (Venken et al., 2006) as the template and domesticating them into *pVD2_purple* via *Bsa*I cloning. The ΦC31 *attB* site containing element was amplified using primers attB-FOR and attB-REV, an element containing the *sopC* and *sopB* genes was amplified using primers moduleA_FOR and moduleA_REV, the element containing *sopA*, *incC* and partial *repE* genes was commercially synthesized and domesticated into *pVD2_purple*, and the element containing the rest of *repE* along with the *oriS* and *oriV* replications of origin was amplified using primers moduleF_FOR and moduleF_REV. All domesticated elements were then assembled with one of four corresponding GB2.0 entry cassettes: *Alpha1*, *Alpha2*, *Omega1* or *Omega2*, and either a kanamycin resistance bacterial marker in the case of Alpha level vectors or a chloramphenicol marker for Omega level vectors using *Bbs*I mediated GB2.0 cloning. Reaction products were transformed, isolated, and analyzed as described above albeit using purple-white colony screening rather than traditional blue-white screening. The resulting *P[acman]* plasmids are totally free of P element transposon inverted transposon repeat sequences and lack a mini-*white* marker, reducing the vector size to just under 7 kb.

Conditionally replicative *pGB2-R6Kγ* GB2.0 compatible vectors were assembled by first PCR amplifying the R6K gamma replication of origin (Rossignol et al., 2001) using primers R6Kγ_FOR and R6Kg_REV from template vector *MCS2-R6Kgamma-CORRECT-Kan-rc* (unpublished data) and domestication of the amplicon into *pVD2* (Sarrion-Perdigones et al., 2013) via a *Bsa*I GB2.0 cloning reaction. *pGB2-R6Kg* vectors were assembled via a *Bbs*I GB2.0 assembly reaction by combining the domesticated *R6Kγ* replication of origin with an appropriate GB2.0 entry cassette (*Alpha1*, *Alpha2*, *Omega1*, or *Omega2*) similar in design to the *pUPD2* entry cassette, and either a domesticated kanamycin or chloramphenicol resistance marker for *Alpha* or *Omega* vectors respectively. A spectinomycin resistance variant of *pGB2-R6Kγ-Alpha1* was also generated as described above but with a spectinomycin resistance marker instead. Resultant assembly products were transformed in *EC100D pir-116* cells (Epicenter/Lucigen), isolated and analyzed as described above.

#### DNA part library and vector toolkit

GoldenBraid 2.0 (GB2.0) DNA parts were designed as follows (for DNA parts see Table S1 and the Key resources table): all non-coding sequences (e.g., promoters, poly(A) terminators, etc.) were synthesized as linear double stranded DNA with appropriate flanking GB2.0 cloning overhangs and restriction enzyme sites (IDT, Eurofins Genomics) or commercially cloned DNA fragments (Twist Bioscience). Internal GB2.0 cloning restriction enzyme sites (*Esp*3I and *Bsa*I) were removed where necessary. All coding DNA sequences were first codon optimized for expression in *Drosophila melanogaster* using an online tool (http://www.idtdna.com/pages/tools/codon-optimization-tool?returnurl=%2FCodonOpt, IDT), then analyzed for splice acceptor and donor sites using a splice site prediction online tool (https://www.fruitfly.org/seq_tools/splice.html, BDGP (Reese et al., 1997), and finally any internal GB2.0 cloning enzyme sites were manually removed prior to commercial DNA synthesis. DNA fragments were domesticated as described above. Initially parts were domesticated into *pUPD* vector backbones (Sarrion-Perdigones et al., 2011). However, due to variable quality of prepared DNA, poor blue-white colony differentiation, and requirement of IPTG induction for strong blue color, we developed *pUPD2* to overcome these limitations. In this vector, DNA parts are insulated from the rest of the vector backbone by flanking pairs of inverted bacterial poly(A) terminator sequences (see above) and LacZ expression is driven by a constitutive synthetic bacterial promoter, Em7 (Zhang et al., 1998). For DNA subcloning, such as when switching the grammar of an already domesticated element, we also developed a third domestication vector, *pUPD3*. This version also features a constitutively expressed LacZ blue-white screening marker and a chloramphenicol bacterial resistance marker to facilitate backbone switching from *pUPD* or *pUPD2*. Smaller parts up to 50 bp were synthesized as a pair of single stranded DNA oligos (Sigma-Aldrich), diluted to 10 μM, mixed in a 1:1 ratio by volume, and allowed to anneal at room temperature for at least an hour. Three μl of annealed oligos was then added to a domestication GB2.0 cloning reaction as described above.

Chemocompetent *DH10B* strain bacterial cells (Invitrogen/Thermo Fisher Scientific) were then transformed via heat shock with 2 μl of assembly product and plated onto solid media containing X-Gal and appropriate antibiotic. Assembly into the *pGB2-R6Kγ* vector backbones was accomplished via electroporation of 1 μl of assembly product into electrocompetent *EC100D pir-116* cells (Epicenter/Lucigen). Assembly into GB2.0 compatible *P[acman]* vector backbones was accomplished by transforming 2 μl of assembly product into chemocompetent *EPI300* cells (Epicenter/Lucigen). Correct assemblies were identified using standard blue-white colony screening. Plasmid DNA was prepared using the QIAprep Spin Miniprep Kit (QIAGEN) for regular high-copy plasmid backbones, and ChargeSwitch-Pro plasmid miniprep kit (Invitrogen/Thermo Fisher Scientific) for *P[acman]* vector backbones, after copy number induction by CopyControl Fosmid Autoinduction Solution (Epicenter/Lucigen, CCIS125). All elements of the DNA part library are summarized in Table S1 and the Key resources table. All primers used are summarized in Table S5 and the Key resources table.

All GB2.0 vectors were cloned using alternating *Bsa*I and *Esp*3I mediated assembly steps as previously described (Sarrion-Perdigones et al., 2013, 2019). Briefly, domesticated parts are initially assembled into transcriptional units (TUs) consisting of, minimally, a promoter, a CDS, and a poly(A) terminator sequence into an Alpha level destination vector via *Bsa*I-HFv2 mediated assembly as described above. More complex assemblies involving additional parts are possible. We are routinely able to assemble up to 7 different parts of varying size and complexity at high efficiency. A pair of Alpha level vectors can then be assembled further using GB2.0 grammar into an Omega level vector via an *Esp*3I mediated assembly reaction to form a pair of TUs. Paired Omega vector can then be assembled again back into an Alpha level vector via *Bsa*I-HFv2 assembly to form a genetic circuit of multiple TUs. This process continues until the desired end product is reached. All cloning backbone vectors, domesticated parts, and assembled plasmids are available through Addgene (https://www.addgene.org/; Tables S1 and S2; Key resources table).

### QUANTIFICATION AND STATISTICAL ANALYSIS

All data were analyzed using Excel (Microsoft Corporation), followed by Prism 7 software (GraphPad Software) for statistical analysis and graphing. Statistical parameters are indicated when applicable at the end of figure legends (Figures 1, S3, and S4). The resulting graphs were then edited for publication using Adobe Illustrator Creative Cloud (Adobe). Immunofluorescent images were obtained as described above and then edited for publication using Adobe Photoshop Creative Cloud and Adobe Illustrator Creative Cloud (Adobe). Statistical methods are described in relevant sections. Interactome analysis was performed and visualized using the biological database and web resource of known and predicted protein–protein interactions, called STRING (Search Tool for the Retrieval of Interacting Genes/Proteins) (https://www.string-db.org; Szklarczyk et al., 2019). Synaptic specific network was further visualized using the open source bioinformatics software platform for visualizing molecular interaction networks, called Cytoscape v3.8.2 (https://cytoscape.org; Shannon et al., 2003).

## Supplementary Material

1

2

3

4

## Figures and Tables

**Table 1.** Summary of selection and counterselection markers

Marker	Species origin	Encoded protein	Reference	Marker size (bp)	Drug(s)^a^	Solvent	Bacterial selection (μg/mL)	Drosophila size ESC/ECC (μg/mL)	Cost ($/vial)

Resistance markers									
*nptII*	*Klebsiella pneumoniae*	neomycin phosphotransferase II	Davies and Smith, 1978	795	kanamycin (bacteria), G418 sulfate (geneticin)	MQ H2O	30	350	0.07
*pac*	*Streptomyces alboniger*	puromycin N-acetyltransferase	Vara et al., 1985	600	puromycin HCl	MQ H2O	100	250–500	2.32– 4.64
*bsr* ^b^	*Bacillus cereus*	blasticidin S-resistance	Itaya et al., 1990	423	blasticidin S	MQ H2O	100	25–45	0.41– 0.73
*hph* ^c^	*Escherichia coli*	hygromycin B phosphotransferase	Gritz and Davies, 1983	1,026	hygromycin B	MQ H2O	75	35–45	0.04– 0.05
*ble* ^d^	*Streptoalleteichus hindustanus*	bleomycin resistance protein	Oliva-Trastoy et al., 2005	375	zeocin or phleomycin	1M HEPES	25 or –	350	– or 9.35
*Sensitivity markers*									
*sr39TK* ^e^	*herpes simplex virus-1*	thymidine kinase	Black et al., 2001	1,131	ganciclovir	0.1N NaOH	–	4	0.01– 0.04
*FCU1* ^e^	*Saccharomyces cerevisiae*	FCU1	Erbs et al., 2000	1,122	5-fluorocytosine	1×PBS	–	10–15	<0.01

The efficacy of seven different markers (five selection and two counterselection) were tested for *in vivo* selection and counterselection in *Drosophila melanogaster*. For each marker, we established an effective selection and counterselection concentration (ESC and ECC) defined as the minimal amount of drug required to either eliminate all non-resistant flies without significantly affecting resistance marker expressing fly viability or to eliminate all sensitivity-marker-expressing flies without significantly affecting non-sensitive flies. For the five selection markers, selection concentrations were also determined for use in bacteria.

a Drugs used to determine cost were G418 sulfate (VWR 97063-060), puromycin HCl (VWR 97064-280), blasticidin S (VWR 71002-676), hygromycin B (VWR AAJ6068103), phleomycin (VWR AAJ67027-8EQ), ganciclovir (TCI America 50-155-694), and 5-fluorocytosine (TCI America 50-014-34810).

b Although basal marker expression is enough to confer effective drug resistance when expressed from tested genomic docking sites, heat-shockenhanced expression is required when inserted into low-expressing genomic loci. Lower marker expression is also associated with blasticidin-S-related toxicity in resistant animals at higher concentrations (≥50 μg/mL).

c Effective hygromycin B resistance requires heat-shock-enhanced expression in all tested contexts.

d Zeocin was found ineffective at selecting flies. Phleomycin is a poorly effective selection agent, with high-ESC, batch-specific variability.

e At higher concentrations, both tested counterselection drugs show general toxicity, with ganciclovir exhibiting toxicity above 15 μg/mL and 5-fluorocytosine above 50 μg/mL.

## Highlights

- *In vivo* analysis of four selection and two counterselection markers
- Individual or multiplexed use of markers to produce complex genotypes
- Demonstrating usefulness of markers through three separate applications
- Provision of DNA and vector resources to facilitate future applications
