# Supplementary material for: Multiplexed drug-based selection and counterselection genetic manipulations in Drosophila
Source: Cell Rep. Author manuscript; Available in PMC 2021 Sep 29. (PMC8480232; doi:10.1016/j.celrep.2021.109700)
Supplement: 1 [file NIHMS1740534-supplement-1.pdf]

Cell Reports, Volume *36*

Supplemental information

Multiplexed drug-based selection and counterselection genetic manipulations in *Drosophila*

Nick Matinyan, Mansi S. Karkhanis, Yezabel Gonzalez, Antrix Jain, Alexander Saltzman, Anna Malovannaya, Alejandro Sarrion-Perdigones, Herman A. Dierick, and Koen J.T. Venken

Cell Reports, Volume *36*

Supplemental information

Multiplexed drug-based selection and counterselection genetic manipulations in *Drosophila*

Nick Matinyan, Mansi S. Karkhanis, Yezabel Gonzalez, Antrix Jain, Alexander Saltzman, Anna Malovannaya, Alejandro Sarrion-Perdigones, Herman A. Dierick, and Koen J.T. Venken

Supplemental figures

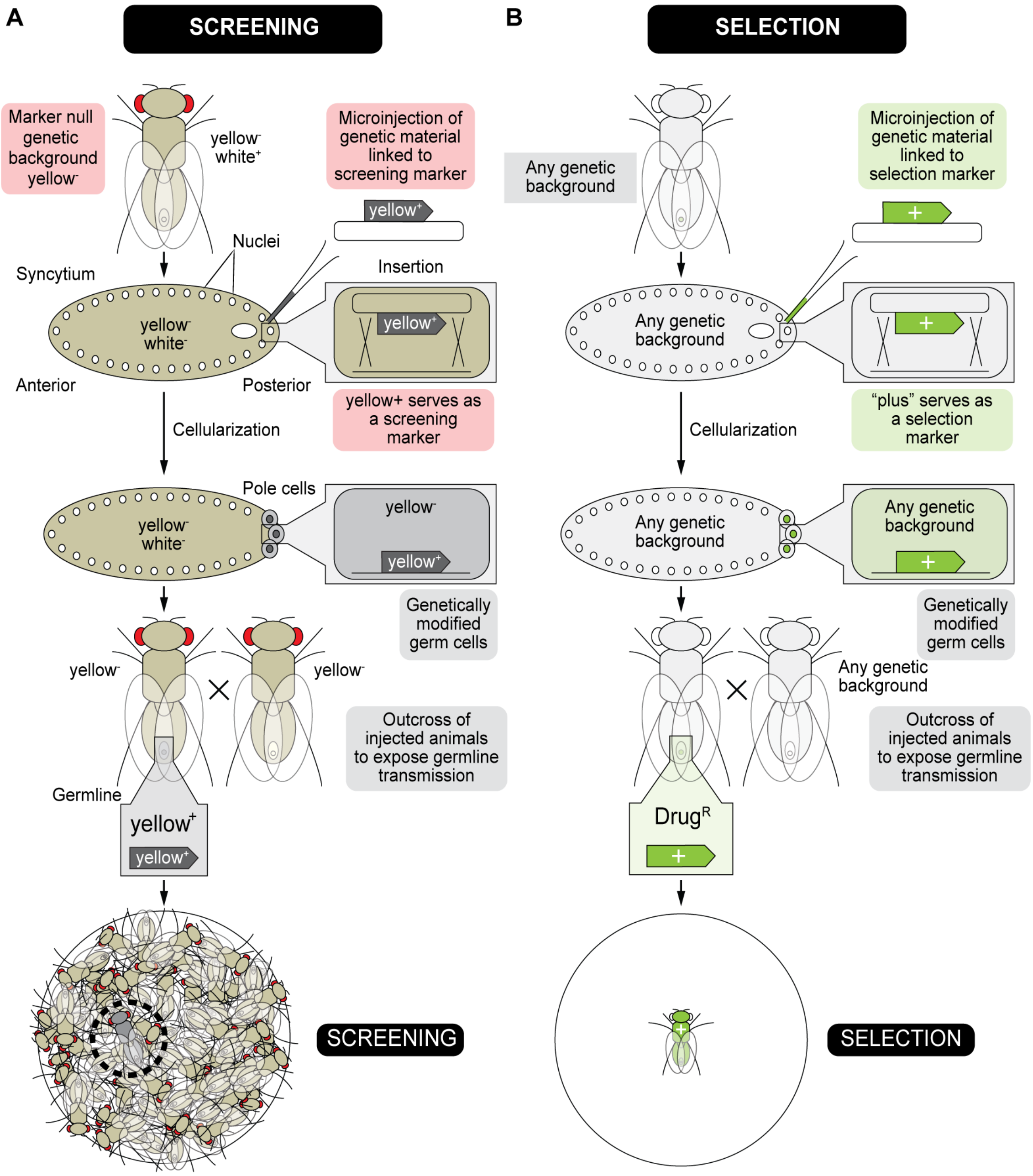

Supplemental figure S1. Comparison of screening with drug-based selection. (**A**) Screening-based tractable germline genetic manipulations rely on dominantly expressed physical markers, such as the *white* gene for eye coloring and the *yellow* gene for body pigmentation, to identify transgenic progeny. Tractable germline genetic manipulations involve microinjection of transgenic or other genetic material, coupled to a physical marker (*yellow*$^{+}$) into the posterior end of early stage embryos targeting the nuclei of the developing germline. Resultant adult animals have a modified germline and must be crossed into a marker deficient, recessive null allele background (*yellow*$^{-}$). Modified progeny are identified by the presence of the marker among otherwise null allele animals. Manual screening can be time consuming depending on the efficiency of transgenesis or another tractable genome engineering paradigm. (**B**) Selection-based tractable germline genetic manipulations instead couple dominantly expressed drug resistance markers (+) to the transgene or other genetic material of interest. Tractable germline selection marker-based genetic manipulations, similarly, involves microinjection of transgenic or other genetic material followed by a cross. However, as selection markers are heterologous to fruit flies, any genetic background can be used in the cross scheme. Progeny are selected on food with drug and only resistant animals survive treatment, eliminating the need to screen modified animals reducing the workload even if transgenesis or another tractable genome engineering paradigm occurs at low frequency. Related to figure 1 and STAR methods.

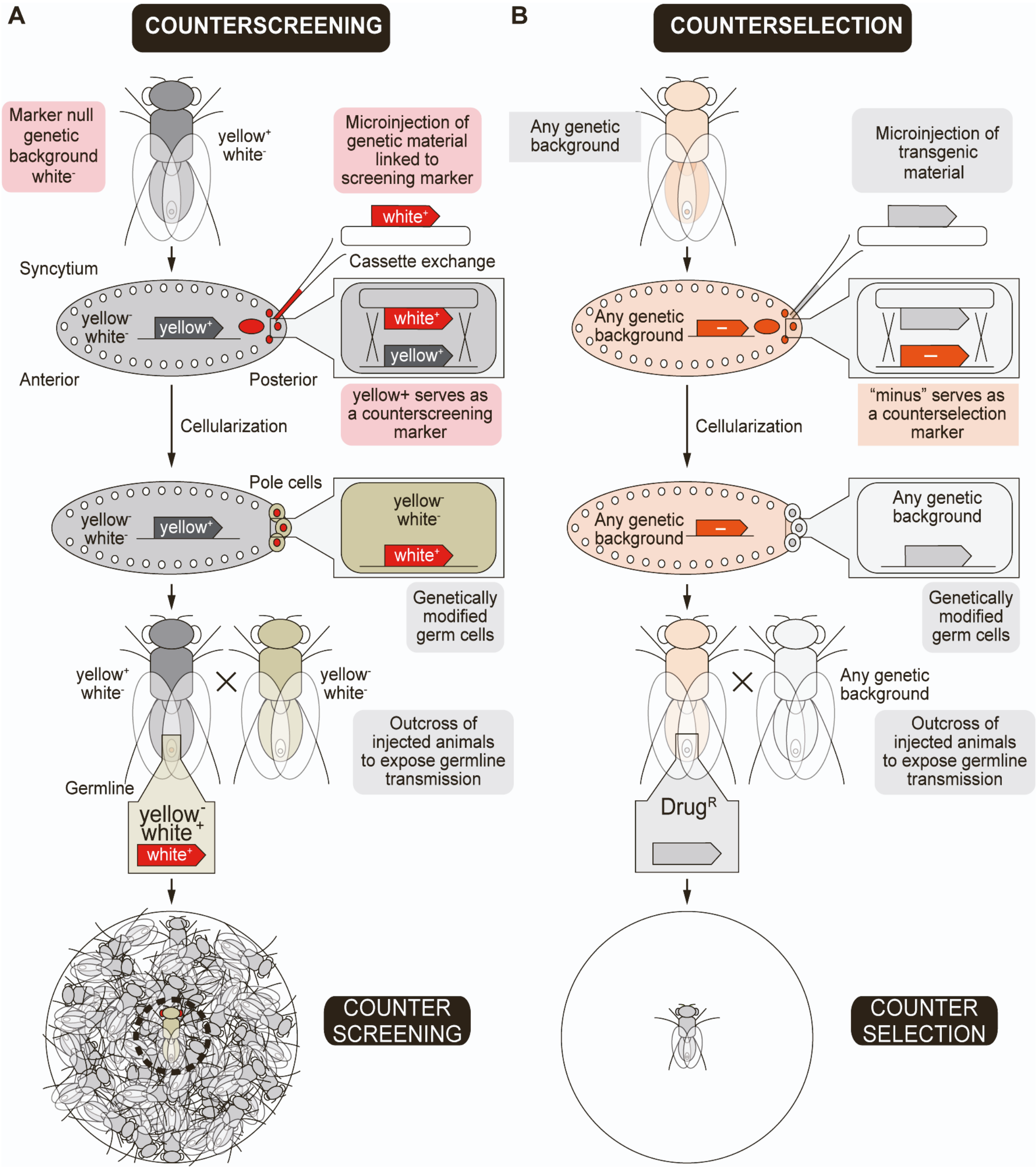

Supplemental figure S2. Comparison of counterscreening with drug-based counterselection. (**A**) Counterscreening involves screening against an undesired physical marker associated with an unwanted genotype. Typically, this involves replacement of the original marker (*yellow*$^{+}$) such as during a recombinase-mediated cassette exchange and then screening for the new marker (*white*$^{+}$) while counterscreening against the original cassette marker in a double recessive null allele background (*yellow*$^{-}$ *white*$^{-}$). (**B**) Counterselection instead couples a drug sensitivity marker (-) to an undesired genotype. Replacement of the marker removes the sensitivity marker allowing desired modified progeny to survive counterselection whereas animals carrying the original cassette retain drug sensitivity. Related to figure 1 and STAR methods.

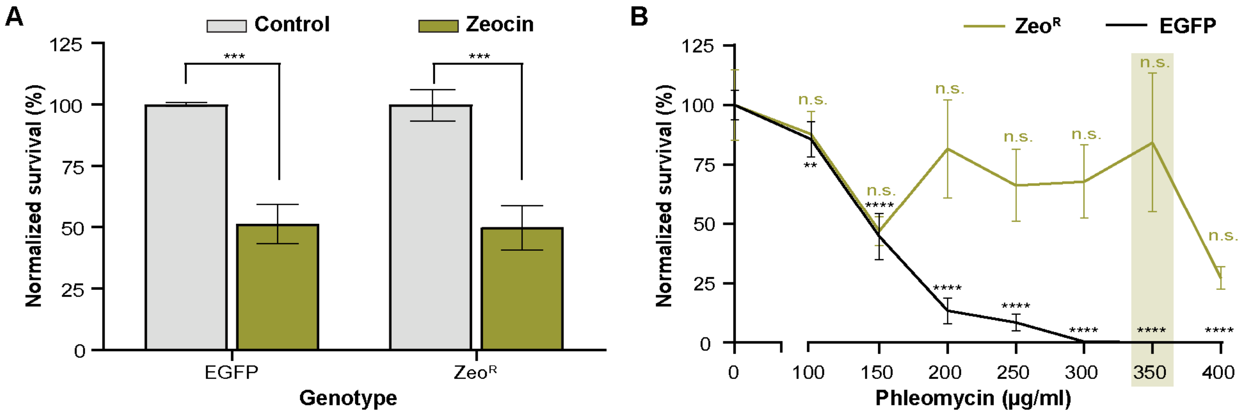

Supplemental figure S3. Zeo^R is a poor selection marker. (**A**) Zeocin, a formulation of phleomycin D1, is ineffective for selection of fruit flies even at a concentration of 1 µg/ml with indistinguishable survival between resistant and control flies. (**B**) Phleomycin is selective for the resistance marker Zeo^R expressing flies but resistance is variable and has poor reliability. Effectiveness of phleomycin selection varies by batch and resistance does not fully inhibit drug-related DNA damage, precluding further testing. Statistical significance was determined via multiple t-test between untreated and treated vials of the same strain for each drug using the Holm-Sidack method (**A**) and Dunnett's multiple comparisons test (**B**) (α = 0.05 *P <0.05, **P < 0.01, ***P < 0.001, and ****P < 0.0001, n.s. is non-significant). Mean and S.E.M. are shown for at least three replicate vials per strain per treatment condition per drug. Related to figure 1.

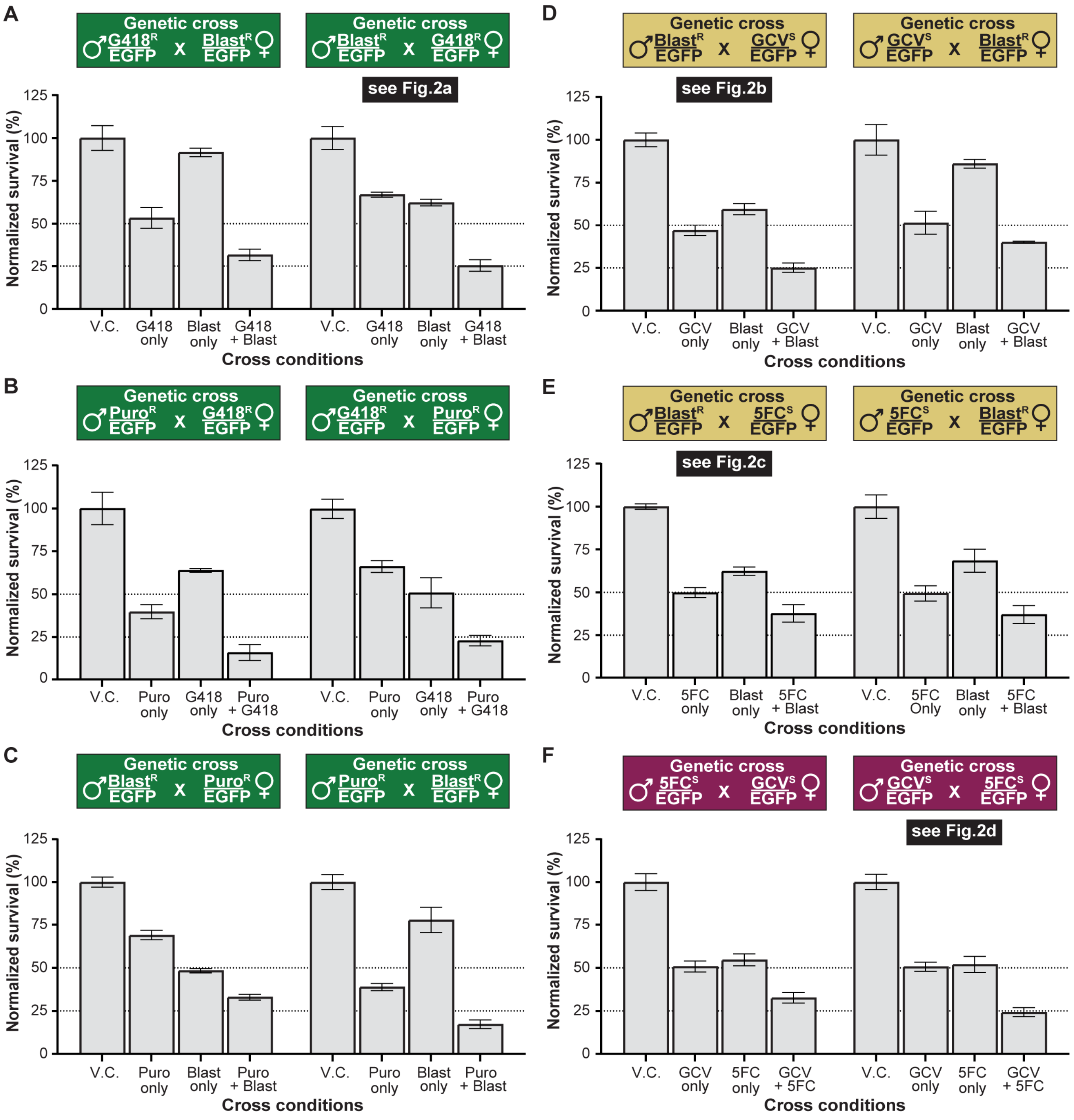

Supplemental figure S4. Drug-based logic gating survival data shows a maternal selection effect. Different resistance marker strain heterozygotes (**A**-**C**), resistance and sensitivity marker heterozygotes (**D**, **E**) and sensitivity marker heterozygotes (**F**) were crossed together under different drug treatment conditions. Flies were crossed either on food with vehicle control, drug 1, drug 2, or drugs 1 and 2 (see STAR Methods). In general we observed survival data that match expected frequencies with single drug treatment reducing survival by 50% and dual drug treatment reducing survival by 75% reflecting selection or counterselection for specific genotypes. However, we did observe a strong maternal selection effect on survival in blasticidin S treated crosses when drug resistance was maternally provided (**A**, **C**, **D,** and **E**). We did not observe a similar effect for female conferred G418 resistance, though there may be a weak maternal selection effect for puromycin resistance (**B**, **C**). Counterselection markers showed no obvious parental effect (**D**-**F**). Differences in survival between drug conditions were obviously large and did not require statistical analysis. Mean and S.E.M. are shown for at least three replicate vials per cross per drug treatment condition. Related to figure 2.

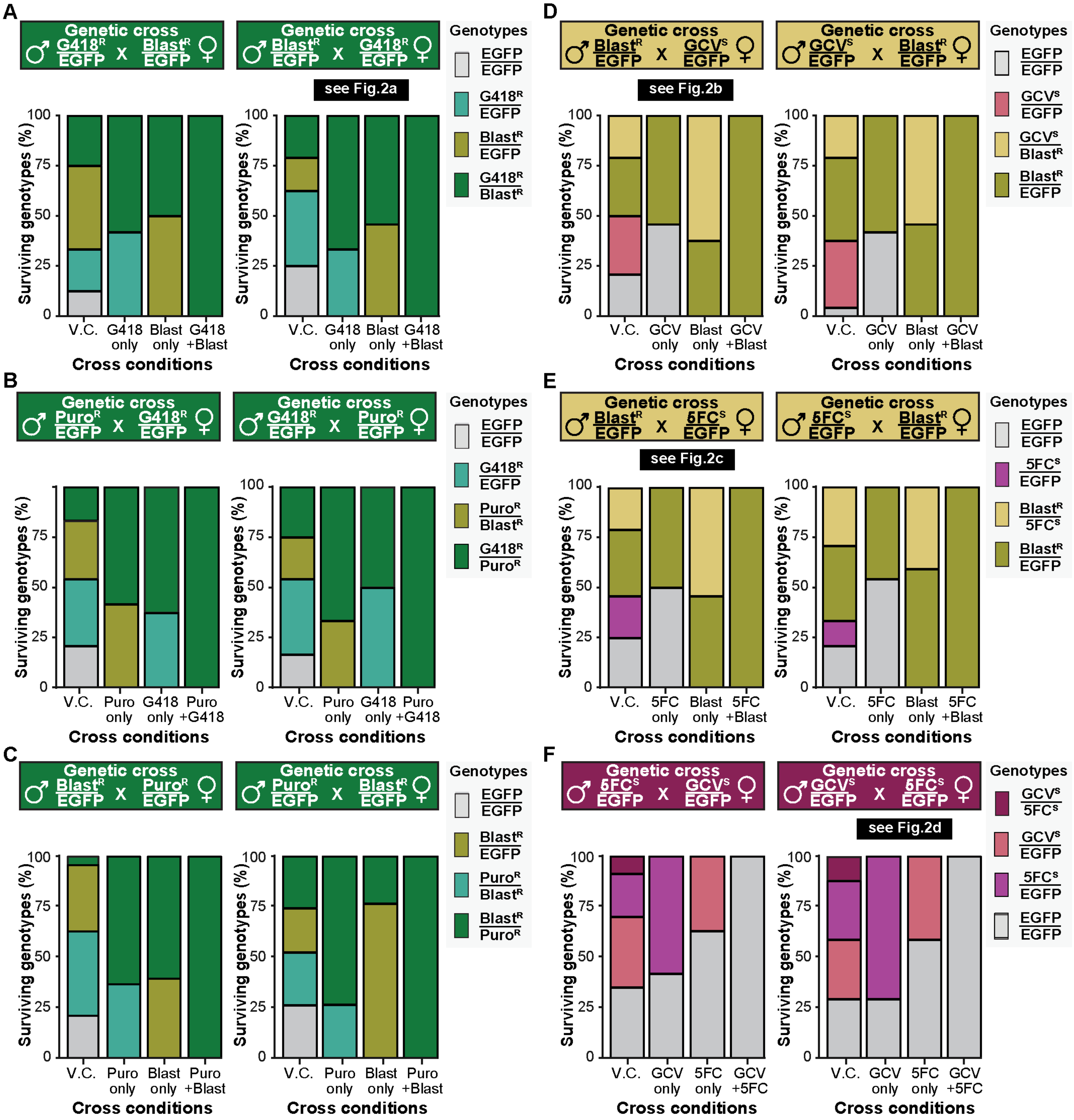

Supplemental figure S5. Genotyping reveals robust dual-drug logic gating regardless of parental origin. For each cross, we genotyped at random 24 flies from each cross condition. In all cross conditions we found only expected genotypes based on the specific selection and/or counterselection conditions for each drug treatment and drug treatment combination (**A**-**F**). Parental (maternal or paternal) origin of the selection marker had no impact on expected genotypes in the resistance drug treatment conditions. Related to figure 2.

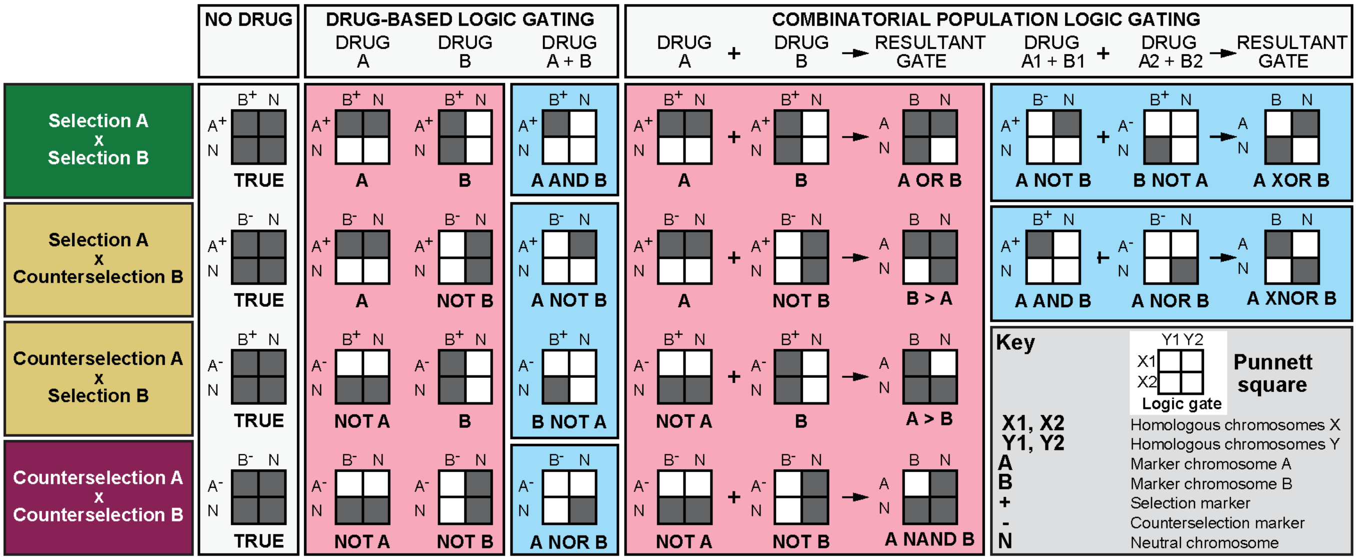

Supplemental figure S6. Drug-based genetic logic gating using paired selection/counterselection markers. There are 16 two-input binary Boolean logic gates, of which eight are commonly used: AND, OR, XOR, NAND, NOR, XNOR, and both versions of NOT. Four of these can be directly represented using multiplexed markers: AND, NOR, and both versions of NOT. If presence of a marker in a genotype is thought of as representing a 1 and its absence a 0 in binary terms, then co-selection of animals from within a mixed, heterozygous population can be seen as representing an AND gate as of the four possible outcomes, only the dual marker expressing transheterozygotes survive drug treatment (A AND B, which is equal to B AND A). Conversely, co-counterselection as described above represents a NOR gate, as only animals without either marker, survive (A NOR B, which is equal to B NOR A). Combination selection and counterselection is akin to a NOT gate with only heterozygotes expressing one marker (selection), but not the other (counterselection, surviving the dual drug treatment (A NOT B or B NOT A). While the other four commonly used logic gates, OR, NAND, XOR, and XNOR, cannot be directly achieved through drug-based selection/counterselection; it is possible to produce them indirectly by combining differently drug-gated populations together. For example, the combination of two single drug selected populations ("A selection gated" plus "B selection gated") results in an OR gate with only the genotype lacking either resistance marker excluded (A OR B, which is equal to B OR A). Conversely, by combining two populations, each singly counterselected with a different drug ("A counterselection gated" plus "B counterselection gated"), it is possible to produce a NAND gate, excluding only the dual sensitivity marker genotype (A NAND B, which equals B NAND A). The addition of two populations, each one treated with both selection and counterselection drugs ("A NOT B" plus "B NOT A"), produces a XOR gate, which excludes genotypes expressing both markers as well as EGFP homozygotes (A XOR B, which equals B XOR A). On the other hand, the addition of a co-selected population to a co-counterselected population ("A AND B" plus "A NOR B") creates a XNOR gate and excludes genotypes expressing either single drug marker genotypes (A XNOR B, which equals B XNOR A). The other eight less used binary Boolean logic gates, some mentioned above already, are indicated as well: A, B, NOT A, NOT B, A > B (B or Not A), B > A (A or Not B), TRUE and FALSE (not shown). Related to figure 2.

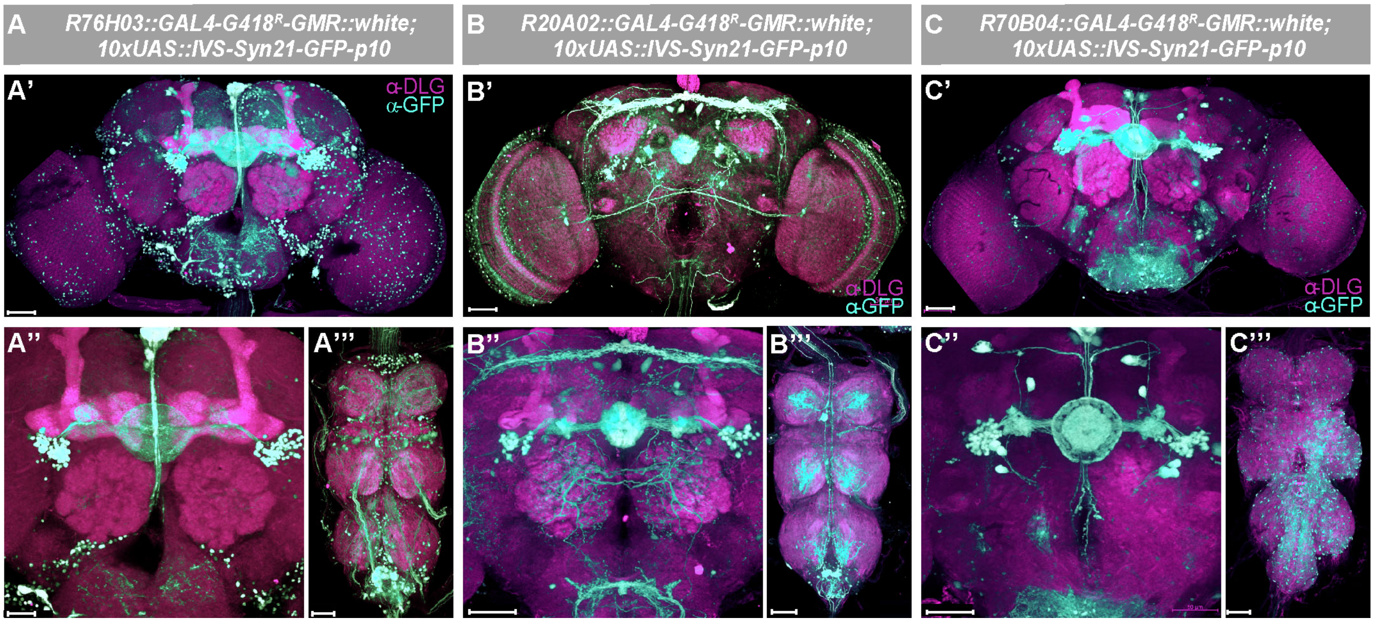

Supplemental figure S7. *GAL4* drivers crossed to a strong *UAS-GFP* reporter line (*JFRC81*) show expression patterns similar to those previously reported. (**A**-**C**) Functionality of GoldenBraid 2.0 cloned *GAL4* drivers were also tested by crossing each driver to an established strong *UAS-GFP* reporter line (*JFCR81*). Enhancer driven expression shows strong similarity to previously reported patterns for all three enhancers: R76H03 (**A**), R20A02 (**B**) and R70B04 (**C**). (**A**) R76H03 driven expression is broader in the adult brain than previously reported (**A'**), albeit staining in the central complex, especially in the ellipsoid body and R4 cells are very similar as previously reported (**A''**). Expression in the VNC is weaker than the previously reported pattern (**A'''**). (**B**) Staining also reveals strong R20A02 driven expression in a posterior dorsal bundle (**B'**) as well as in the central complex (**B''**) of the adult brain. R20A02 expression in the ventral nerve cord (VNC) is distinct from previously reported enhancer-GAL4 labeling (**B'''**). (**C**) R70B04 driven expression shows strong similarity to previous reports in the adult brain (**C'**), central complex (**C''**) and the VNC (**C'''**). Differences in expression patterns are likely due to the use of different genomic docking sites and vector design that may affect expression levels in different cell subpopulations (see STAR Methods for details on immunofluorescence). Scale bars represent 50 μm. Related to figure 3.

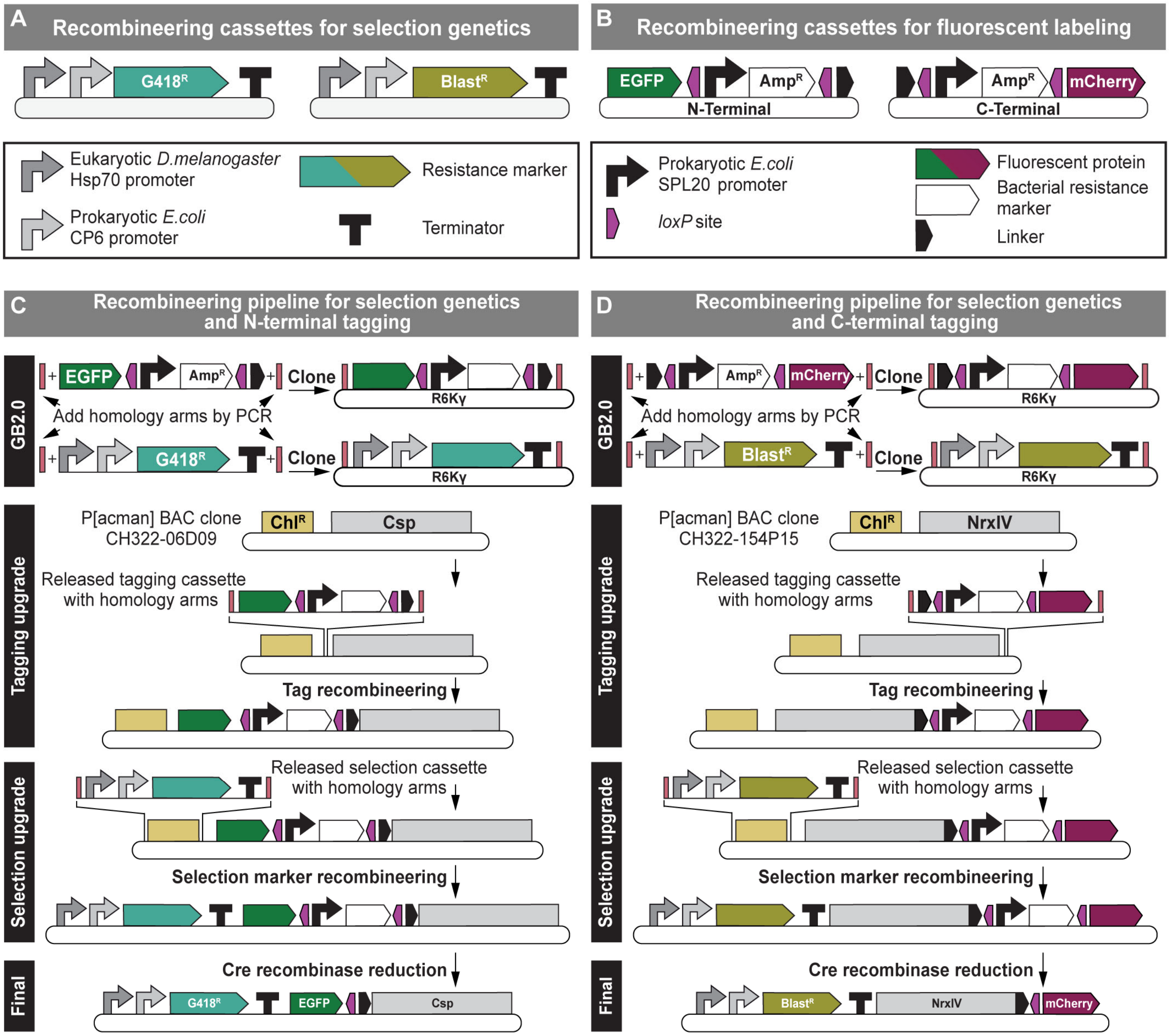

Supplemental figure S8. Detailed schematic of three-step serial recombineering strategies for upgrading of genomic *P[acman]* BAC clones. (**A**-**B**) Using GoldenBraid 2.0 cloning (GB2.0), we generated recombineering cassettes for upgrading genomic *P[acman]* BAC clones to be orthogonally drug selectable in the fruit fly, *i.e.*, G418^R or Blast^R (**A**), and tagging them with a fluorescent protein either on the N- or C-terminus (**B**). (**C**, **D**) Cassettes were amplified via PCR using overhang primers to add BAC specific 50 bp homology arms and then GB2.0 cloned into compatible conditionally replicative vector backbones (*R6Kγ*). BACs were upgraded via serial recombineering by electroporating restriction enzyme linearized tagging and selection marker cassettes into *SW102* bacterial cells. Induction of Cre recombinase reduces the ampicillin marker producing the final selectable and fluorescently N-terminally (**c**) or C-terminally (**d**) tagged *P[acman]* BAC clone. See STAR Methods for further details. Related to figure 5.

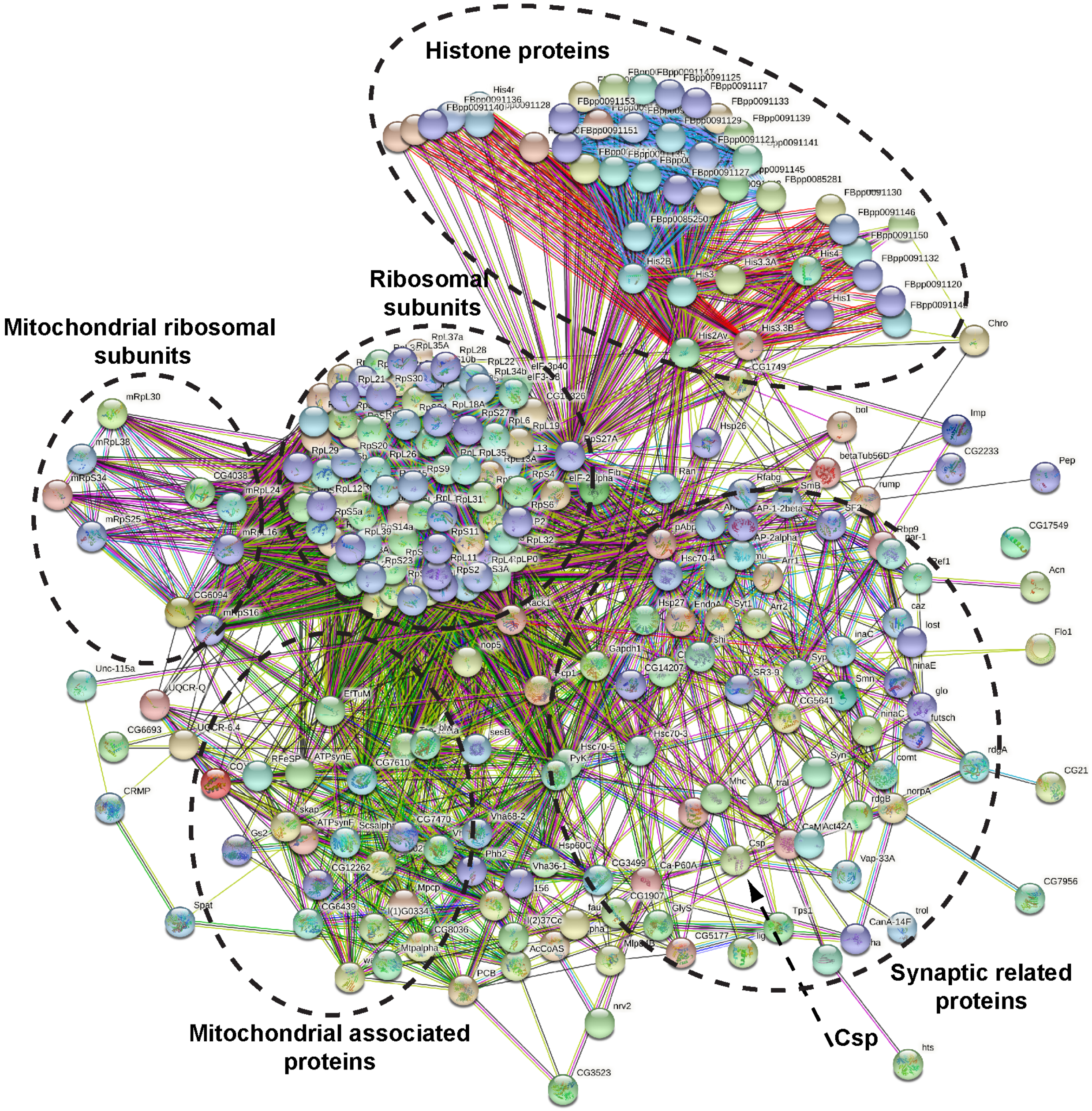

Supplemental Figure S9. Whole Csp interactome from tagged Csp pulldown and mass spectrometry analysis. Visualization of the protein-protein interactome based on unbiased proteomic analysis of N-terminally, EGFP tagged Csp pulldown versus EGFP control. Interactome analysis and visualization was performed using the database of known and predicted protein-protein interactions, called STRING (STRING v11, www.string-db.org). Various related protein groups are highlighted in the visualization. Related to figure 5.

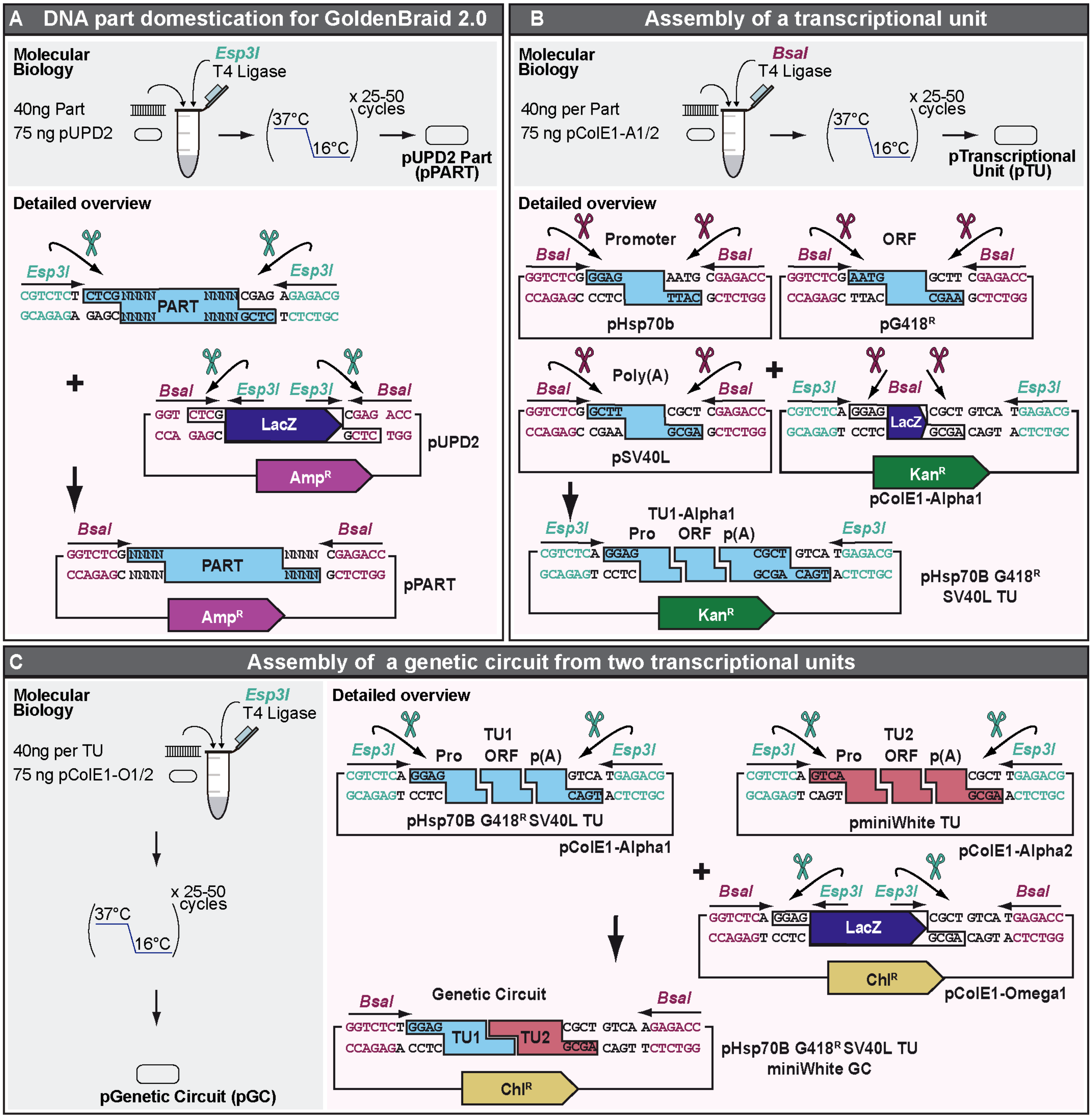

Supplemental figure S10. Detailed schematic of GoldenBraid 2.0 DNA part domestication and iterative assembly. (**A**) Integration, or domestication, of novel parts into GB2.0 begins with synthesis or PCR amplification of a part to include GB2.0 cloning overhangs as well as requisite *Esp*3I restriction enzyme binding sites for assembly, into a "plasmid Universal Part Domesticator (*pUPD*)" vector. (**B**) Once domesticated, multiple parts can be assembled together into a functional transcriptional unit (TU) in a one-pot, *Bsa*I mediated cloning reaction into "Alpha" level vectors (*pColE1-Alpha1* or *pColE1-Alpha2*). For example, a G418 resistance TU, consisting of promoter, open reading frame (ORF), and polyadenylation signal (p(A)) can be assembled into a destination vector (*pColE1-Alpha1*) in a single assembly step. (**C**) Pairs of TUs, for example a G418 resistance TU and a mini-white eye marker TU, can then be further assembled to form a genetic circuit of multiple TUs through assembly into an "Omega" level vector (*pColE1-Omega1* or *pColE1-Omega2*) via *Esp*3I mediated reaction. Assembly of TUs is directional with a TU in pColE1-Alpha/Omega1 always joining to the 5' overhang of the destination vector backbone and the TU in *pColE1-Alpha2/Omega2* joining to the 3' destination overhang. Importantly the products of one GB2.0 reaction can be used as reagents for further assembly, allowing for the iterative assembly of complex constructs of multiple circuits via alternating *Bsa*I/*Esp*3I assembly reactions. Related to figure 6.

Supplemental tables

Fly strain genotype	Abbreviated name	Description	BDSC #
y[1]w[1118]; PBac{y[+mDint2] w[+mC]=P[acman]-attB-Hsp70-CP6EGFP-}VK00033	*EGFP*	EGFP expressing control strain	Cat# 92331, RRID:BDSC_92331
y[1]w[1118]; PBac{y[+mDint2] w[+mC]=P[acman]-attB-Hsp70-CP6G418R-}VK00033	*$G418^R$*	G418 resistant stock	Cat# 92332, RRID:BDSC_92332
y[1]w[1118]; PBac{y[+mDint2] w[+mC]=P[acman]-attB-Hsp70-CP6-PuroR}VK00033	*$Puro^R$*	Puromycin resistant stock	Cat# 92333, RRID:BDSC_92333
y[1]w[1118]; PBac{y[+mDint2] w[+mC]=P[acman]-attB-Hsp70-CP6-BlastR}VK00033/TM6B, Tb[1]	*$Blast^R$*	Blasticidin resistant stock	Cat# 92334, RRID:BDSC_92334
y[1]w[1118]; PBac{y[+mDint2] w[+mC]=P[acman]-attB-Hsp70-CP6-HygroR}VK00033	*$Hygro^R$*	Hygromycin resistant stock	Cat# 92335, RRID:BDSC_92335
y[1]w[1118]; PBac{y[+mDint2] w[+mC]=P[acman]-attB-Hsp70-CP6-ZeoR}VK00033	*Zeo^R*	Phleomycin resistant stock	Cat# 92336, RRID:BDSC_92336
y[1]w[1118]; PBac{y[+mDint2] w[+mC]=P[acman]-attB-Hsp70-CP6GCVS}VK00033	*GCV^S*	Ganciclovir and acyclovir sensitive stock	Cat# 92337, RRID:BDSC_92337
y[1]w[1118]; PBac{y[+mDint2] w[+mC]=P[acman]-attB-Hsp70-CP6-5FCS-TKpA}VK00033	*$5FC^S$*	5-fluorocytosine sensitive stock	Cat# 92338, RRID:BDSC_92338
P{w[+mW.Scer\FRT.hs]=RS3}l(1)CB-6411-3[1], w[1118]/FM7h, P{UAS.RMCE.w[+]}1B	*FM7R-25C-1B*	FM7R balancer chromosome stock with P-element insertion of a ΦC31 RMCE cassette	Cat# 92339, RRID:BDSC_92339
y[1] w[1118]/Dp(1;Y)y[+]; L[1]/CyO, P{UAS.RMCE.w[+]}4A	*CyO-25C-4A*	CyO balancer chromosome stock with P-element insertion of a ΦC31 RMCE cassette	Cat# 92340, RRID:BDSC_92340
y[1] w[1118]/Dp(1;Y)y[+]; L[1]/CyO, P{UAS.RMCE.w[+]}3A	*CyO-52D-3A*	CyO balancer chromosome stock with P-element insertion of a ΦC31 RMCE cassette	Cat# 92341, RRID:BDSC_92341
y[1] w[1118]/Dp(1;Y)y[+]; D[1]/TM6B, P{UAS.RMCE.w[+]}5A, Tb[1]	*TM6B-25C-5A*	TM6b,TB balancer chromosome stock with P-element insertion of a ΦC31 RMCE cassette	Cat# 92342, RRID:BDSC_92342
y[1] w[1118]/Dp(1;Y)y[+]; D[1]/TM6B, P{UAS.RMCE.w[+]}1A, Tb[1]	*TM6B-52D-1A*	TM6b,TB balancer chromosome stock with P-element insertion of a ΦC31 RMCE cassette	Cat# 92343, RRID:BDSC_92343
y[1] w[1118]/Dp(1;Y)y[+]; D[1]/TM6B, P{BlastR.5FCS.w[+]}5A, Tb[1]	*$TM6b^{Tb}$::$Blast^R$$5FC^S$*	TM6B, Tb blasticidin resistant, 5-fluorocytosine sensitive balancer stock	Cat# 92344, RRID:BDSC_92344
y[1] w[1118]/Dp(1;Y)y[+]; D[1]/TM6B, P{G418R.5FCS.w[+]}5A, Tb[1]	*$TM6b^{Tb}$::$G418^R$$5FC^S$*	TM6, Tb G418 resistant, 5-fluorocytosine sensitive balancer stock	Cat# 92345, RRID:BDSC_92345
y[1] M{RFP[3xP3.PB] GFP[E.3xP3]=vas-int.B}ZH-2A w[*]; PBac{y[+]-attP-3B}VK00033, PBac{y[+]-attP-9A}VK00020	*2xattP::VK00033;VK00020*	Double genomic docking site fly stock	Cat# 92346, RRID:BDSC_92346
y[1] w[1118]; PBac{y[+mDint2] w[+mC]=CH322-06D09-N-EGFP-Csp.G418}VK00033	*N-EGFP-Csp*	EGFP N-terminally tagged Cysteine string protein BAC stock	Cat# 92347, RRID:BDSC_92347
y[1] w[1118]; PBac{y[+mDint2] w[+mC]=CH322-154P15-NrxIV-C-mCh.BlastR}VK00033	*NrxIV-C-mCherry*	mCherry C-terminally tagged Cysteine string protein BAC stock	Cat# 92348, RRID:BDSC_92348
w[5xGMR-CDS]=pR20A02-GAL4-$G418^R$ }VK000XX, w[5xGMR-CDS]=p5xUAS-sfGFP-$Blast^R$ }VK000XX	*R20A02::GAL4; 5xUAS::sfGFP*	GAL4/UAS binary system dual transgenic; R20A02 enhancer driven GAL4/UAS using sfGFP reporter	Cat# 92764, RRID:BDSC_92364
w[5xGMR-CDS]=pR76H03-GAL4-$G418^R$ }VK000XX, w[5xGMR-CDS]=p5xUAS-mCherry-$Blast^R$ }VK000XX	*R76H03::GAL4; 5xUAS::mCherry*	GAL4/UAS binary system dual transgenic; R76H03 enhancer driven GAL4/UAS using mCherry reporter	Cat# 92765, RRID:BDSC_92365

w[+mC]=pR70B04-LexA-G418^R }VK000XX, w[+mC]=p12xLexAOp-sfGFP-Blast^R }VK000XX	*R70B04::LexA; 12xLexAOp::sfGFP*	LexA/LexaOp binary system dual transgenic; R70B04 enhancer driven LexA/LexAOp using sfGFP reporter	Cat# 92766, RRID:BDSC_92366
y[1] w[67c23]	*IsoY^1*	Yellow-, white- isogenized stock (chromosomes 1, 2 and 3 only)	Cat# 92349, RRID:BDSC_92349

Supplemental table S3. Generated fly stocks. Novel fly stocks generated during the course of this work. All stocks are available through the Bloomington Drosophila Stock Center (https://bdsc.indiana.edu/). Research Resource Identifiers, RRID. See STAR Methods for detailed description on the generation of fly strains. Related to STAR Methods.

Primer type	Primer name	Primer sequence
Regular	Hsp70-F	AAGGAAAAAAGCGGCCGCCTAGAATCCCAAAACAAACTG
	Hsp70-CP6-R	GAATAAACTCCCACATGGATTCGTATTCAGAGTTCTCTTCTTG
	Hsp70-CP6-F	CAAGAAGAGAACTCTGAATACGAATCCATGTGGGAGTTTATTC
	CP6-G418-R	CAATCCATCTTGTTCAATCATTTTGTTTTCCTCCTTATGTTAAG
	CP6-G418-F	CTTAACATAAGGAGGAAAACAAAATGATTGAACAAGATGGATTG
	HSVTK-R1	AACAAACGACCCAACACCCGTGCGTTTTATTCTGTCTTTTTATTGCCTCAGAAGAACTCGTCAAGAAGG
	HSVTK-R2	AAGGAAAAAAGCGGCCGCGAACAAACGACCCAACACCCGTG
SLIC	EGFP-F	GCTTAACATAAGGAGGAAAACAAAATGGTGAGCAAGGGCGAGGAG
	EGFP-R	GTTTTATTCTGTCTTTTTATTGCCTTACTTGTACAGCTCGTC
	G418-F	GCTTAACATAAGGAGGAAAACAAAATGATCGAGCAGGATGGACTG
	G418-R	GTTTTATTCTGTCTTTTTATTGCCTTAGAAGAACTCGTCCAGCAG
	Puro-F	GCTTAACATAAGGAGGAAAACAAAATGACCGAGTACAAGCCCACGGTG
	Puro-R	GTTTTATTCTGTCTTTTTATTGCCTTAGGCGCCTGGCTTGCGGGTCATG
	Blast-F	GCTTAACATAAGGAGGAAAACAAAATGAAGACCTTCAACATCAG
	Blast-R	GTTTTATTCTGTCTTTTTATTGCCTTAGTTGCGGGTGTACTTCAG
	Hygro-F	GCTTAACATAAGGAGGAAAACAAAATGAAGAAGCCCGAGCTGAC
	Hygro-R	GTTTTATTCTGTCTTTTTATTGCCTTACTCCTTGGCGCGTGGGCGGGTG
	Zeo-F	GCTTAACATAAGGAGGAAAACAAAATGGCCAAGCTGACCAGCGCCGTG
	Zeo-R	GTTTTATTCTGTCTTTTTATTGCCTTAATCCTGCTCCTCGGCCACGAAG
	GCV-F	GCTTAACATAAGGAGGAAAACAAAATGGCCAGCTACCCCTGCCATC
	GCV-R	GTTTTATTCTGTCTTTTTATTGCCTTAATTAGCCTCGCCCATCTCGCGGGCGAAG
	5FC-F	GCTTAACATAAGGAGGAAAACAAAATGGTGACCGGCGGCATGGCCAG

	5FC-R	GTTTTATTCTGTCTTTTTATTGCCTTACACGCAGTAGTAGCGATC
**Genotyping**	EGFP-SEQ	CGTAAACGGCCACAAGTTCAG
	TKpA-SEQ	GAACAACGACCCAACACCC
	G418-SEQ	GCTGTTCGTCAAGACCGATC
	Puro-SEQ1	TTAATTAAGAACAAACGACCCAACACCC
	Puro-SEQ2	CGCCCCACGCAATCTGCCCTTCTACGAG
	5FC-SEQ	CAGGGTGGTATCCTTGTACACC
	Blast-SEQ	AGGATCTGGAACTGGTGGAG
	VK33_RIGHT_F	GCATGCGTCAATTTTACGCAG
	VK33_RIGHT_R	TTGCCTGTGACAGCGACATG
	VK33_LEFT_F	TTTTCCCTGCCACCCCTTTAC
	VK33_LEFT_R	CGCATGATTATCTTTAACGTACGTCAC
	VK20_RIGHT_F	CGGCGACTGAGATGTCCTAAATG
	VK20_RIGHT_R	GCGAGTTGGACTTAGCCGAG
	VK20_LEFT_F	CAAAGAGCTTGTCGCTGCAC
	VK20_LEFT_R	CGCATGATTATCTTTAACGTACGTCAC
**GB2.0 cloning**	TKpA-F	CTCGGCTTGGCAATAAAAAGACAGAATAAAACGCACGGGTGTTGGGTCGTTTGTTCCGCT
	TKpA-R	CTCGAGCGGAACAAACGACCCAACACCCGTGCGTTTTATTCTGTCTTTTTATTGCCAAGC
	attB-FOR	CGCGGGTCTCAACTCTGACGAATTGGTCGACGATGTAGG
	attB-REV	CGCGGGTCTCAACTCGAAGCATCATGATGGACCAGATGG
	ModuleA-FOR	CGCGGGTCTCAACTCCTTCTAAGCAGGACACAGCAGCAA
	ModuleA-REV	CGCGGGTCTCAACTCGTCGCGTGTTCCAACTGAGTG
	ModuleF-FOR	CGCGGGTCTCAACTCGACACGATGCCTGAGCCATC

	ModuleF-REV	CGCGGGTCTCAACTCTCACCGCCGCAGTCGAGCGACAG
	R6Kg-FOR	CGCGGGTCTCAACTCTGACGTACCGCGGCCGCGTAGAG
	R6Kg-REV	CGCGGGTCTCAACTCTCACATGTCAGCCGTTAAGTGTTCC
**Recombineering**	Marker-RECO1-F	CTGGTGTCCCTGTTGATACCGGGAAGCCCTGGGCCAACTTTTGGCGAAACTAGAATCCCAAAACAAACTG
	Marker-RECO1-R	GCGTAGCAACCAGGCGTTTAAGGGCACCAATAACTGCCTTAAAAAAAGAACAAACGACCCAACACCCGTG
	Marker-RECO2-F	CGCGGGTCTCTGGAGTCTAGACTGGTGTCCCTGTTGATACC
	Marker-RECO2-R	CGCGGGTCTCTAGCGTCTAGAGCGTAGCAACCAGGCGTTTAAGG
	Csp-RECO1-F	ATCGCTAGTGCAAGTTACCCGTTCGCAGTCAAAGTGACACAGGCATCAGGATGGTGAGCAAGGGCGAGGAG
	Csp-RECO1-R	GCACTACAAAATACTTACGACAGTTTTCTCTTGTCCATGCCAGGTGCGCTgCTGCCGCCGCTACCTCC
	Csp-RECO2-F	CGCGGGTCTCTGGAGCTCGAGATCGCTAGTGCAAGTTACCCGTTCG
	Csp-RECO2-R	CGCGGGTCTCTCGCGCTCGAGGCACTACAAAATACTTACGACAG
	Nrx-RECO1-F	TGCACTCAACTACTGGCCATCAAGTCAGGAAGCGAACAGAGATCTTTATCGGAGGTTCCGGTGGAAGC
	Nrx-RECO1-R	CCTAAGGCGATTTAAAAACGATTTATGTGCATATTCTTGCGACCGGTTTACTTGTACAGCTCATCCATGC
	Nrx-RECO2-F	CGCGGGTCTCTGGAGCTCGAGTGCACTCAACTACTGGCCATCAAGTCAGG
	Nrx-RECO2-R	CGCGGGTCTCTAGCGCTCGAGCCTAAGGCGATTTAAAAACGATTTATGTGC

Supplementary Table S5. Cloning and genotyping primers. All primers and their sequences used during the course of this work, categorized according to their usage. Related to STAR Methods.
